# Nitrogen Cycling from Increased Soil Organic Carbon Contributes Both Positively and Negatively to Ecosystem Services in Wheat Agro-Ecosystems

**DOI:** 10.3389/fpls.2017.00731

**Published:** 2017-05-10

**Authors:** Jeda Palmer, Peter J. Thorburn, Jody S. Biggs, Estelle J. Dominati, Merv E. Probert, Elizabeth A. Meier, Neil I. Huth, Mike Dodd, Val Snow, Joshua R. Larsen, William J. Parton

**Affiliations:** ^1^Queensland Bioscience Precinct, CSIROSt Lucia, QLD, Australia; ^2^AgResearch, Grasslands Research CentrePalmerston North, New Zealand; ^3^CSIROToowoomba, QLD, Australia; ^4^AgResearch, Lincoln Research CentreLincoln, New Zealand; ^5^School of Earth and Environmental Sciences, University of QueenslandSt Lucia, QLD, Australia; ^6^Institute for Earth Surface Dynamics, University of LausanneLausanne, Switzerland; ^7^Natural Resource Ecology Laboratory, Colorado State UniversityFort Collins, CO, USA

**Keywords:** Soil organic matter, modelling, agriculture, drained upper limit, lower limit, plant available water

## Abstract

Soil organic carbon (SOC) is an important and manageable property of soils that impacts on multiple ecosystem services through its effect on soil processes such as nitrogen (N) cycling and soil physical properties. There is considerable interest in increasing SOC concentration in agro-ecosystems worldwide. In some agro-ecosystems, increased SOC has been found to enhance the provision of ecosystem services such as the provision of food. However, increased SOC may increase the environmental footprint of some agro-ecosystems, for example by increasing nitrous oxide emissions. Given this uncertainty, progress is needed in quantifying the impact of increased SOC concentration on agro-ecosystems. Increased SOC concentration affects both N cycling and soil physical properties (i.e., water holding capacity). Thus, the aim of this study was to quantify the contribution, both positive and negative, of increased SOC concentration on ecosystem services provided by wheat agro-ecosystems. We used the Agricultural Production Systems sIMulator (APSIM) to represent the effect of increased SOC concentration on N cycling and soil physical properties, and used model outputs as proxies for multiple ecosystem services from wheat production agro-ecosystems at seven locations around the world. Under increased SOC, we found that N cycling had a larger effect on a range of ecosystem services (food provision, filtering of N, and nitrous oxide regulation) than soil physical properties. We predicted that food provision in these agro-ecosystems could be significantly increased by increased SOC concentration when N supply is limiting. Conversely, we predicted no significant benefit to food production from increasing SOC when soil N supply (from fertiliser and soil N stocks) is not limiting. The effect of increasing SOC on N cycling also led to significantly higher nitrous oxide emissions, although the relative increase was small. We also found that N losses via deep drainage were minimally affected by increased SOC in the dryland agro-ecosystems studied, but increased in the irrigated agro-ecosystem. Therefore, we show that under increased SOC concentration, N cycling contributes both positively and negatively to ecosystem services depending on supply, while the effects on soil physical properties are negligible.

## Introduction

Soils provide multiple ecosystem services that meet human needs (Robinson et al., 2014). In agro-ecosystems these services include both provisioning services, such as food production, and regulating services, such as filtering of nutrients (Millennium Ecosystem Assessment, 2005; Dominati et al., 2010). Soils are therefore a critical natural capital stock (Dominati et al., 2016). The characteristics of soils that influence their capacity to provide ecosystem services can either be inherent or manageable (Dominati et al., 2010). Inherent soil properties, such as soil texture, result from soil formation conditions, and change little over timescales of hundreds of years. Manageable properties, such as organic carbon content or pH, are those more easily modified by management or natural variability on shorter timescales. Land use and management primarily impact these manageable characteristics of soils, and through this the capacity of soils to contribute to ecosystem services provision.

As a manageable property, soil organic carbon (SOC) contributes to ecosystem services through its effect on multiple soil processes and functions. Soil organic carbon (SOC) affects nutrient cycling and soil fertility status. Decomposition of soil organic matter releases nutrients, including nitrogen (N), into soil (Havlin et al., 1990; Hoyle et al., 2011; Murphy, 2015). Thus, a soil with a higher SOC concentration results in a greater the release of organic N to the soil than a soil with a lower SOC concentration (Aggarwal et al., 1997; Kusumo et al., 2011; Murphy, 2015). In addition, SOC affects multiple soil physical properties. An increase in SOC concentration decreases bulk density (Adams, 1973; Manrique and Jones, 1991; Tranter et al., 2007), generally increases soil water holding capacity (Vereecken et al., 1989; Wosten et al., 1999; Saxton and Rawls, 2006) and has a variable effect on hydraulic conductivity (Vereecken et al., 1990; Saxton and Rawls, 2006; Weynants et al., 2009). Many agricultural soils have been significantly depleted of SOC stocks (Cole et al., 1993; Davidson and Ackerman, 1993; Lal, 2004). Therefore, there is considerable interest in increasing SOC concentrations in agro-ecosystems globally to both sequester carbon for climate change mitigation and improve soil quality to enhance productivity and agro-ecosystem sustainability (Reeves, 1997; Post and Kwon, 2000; Lal, 2004; Smith, 2008). These reflect improvements in key desirable ecosystem services, regulating (e.g., carbon sequestration) and provisioning (e.g., crop productivity). However, in order to better inform investments for increasing SOC, it is essential to test and quantify how increased SOC concentration is likely to contribute to various ecosystem services across a range of agro-ecosystems.

Food production is an ecosystem service that is affected by SOC concentration. Increased SOC concentration have been linked with a direct increase in food production; although this varies with land use, soil type, environmental conditions, and management practice (Barzegar et al., 2002; Lal, 2006; Zhang et al., 2012). Other ecosystem services affected by SOC are the regulating services of flood mitigation and water recharge (Dominati et al., 2014). These services are related to the infiltration into, storage in and transmission of water through the soil profile, and so the role of SOC in increasing soil water storage (Gupta and Larson, 1979; Hudson, 1994; Saxton and Rawls, 2006) and changing soil hydraulic conductivity (Vereecken et al., 1990; Saxton and Rawls, 2006) is important in determining the provision of these services.

While increased SOC concentrations can positively influence ecosystem services deemed beneficial, increased SOC may also increase the environmental footprint of a given agro-ecosystem. The increase in organic N associated with an increase in SOC concentration (Hoyle et al., 2011; Murphy, 2015) influences the filtering of N ecosystem service, which refers to capacity of soils to store and retain N (Dominati et al., 2014). While this increase in soil N can be beneficial for crop growth, it can increase net losses of N from agro-ecosystems to ground water aquifers or rivers (Beckwith et al., 1998; Knappe et al., 2002) and have negative environmental impacts such as eutrophication in downstream water bodies (Carpenter et al., 1998). Furthermore, increased SOC can also increase nitrous oxide emissions from agro-ecosystems (Qiu et al., 2009; Burgin et al., 2013).

Quantifying both the positive and negative effects of SOC on the provision of ecosystem services is essential to provide a more comprehensive understanding of the impact of SOC on agro-ecosystems (e.g., Swinton et al., 2007; Zhang et al., 2007; Power, 2010). However, few studies have quantified the effect of SOC on multiple ecosystem services. Ghaley and Porter (2014) found that increased SOC concentration in a wheat production system increased food production and carbon sequestration for a site in Denmark, but did not quantify impacts on other ecosystem services such as nitrous oxide regulation. Balbi et al. (2015) found that reductions in manure application to cropping systems in Spain provided the ecosystem service benefit of reducing loss of N to the environment at the expense of yield and carbon sequestration. Furthermore, as described above, increased SOC concentration affects both N cycling (the store of N in soil) and soil physical properties (e.g., soil water holding capacity). However, the relative contribution of these soil attributes to ecosystem services provision has not been quantified previously for multiple ecosystem services across a range of agro-ecosystems.

We hypothesised that the effect of increased SOC concentration on both N cycling and soil physical properties (e.g., influencing soil water holding capacity) would contribute both positively and negatively to ecosystem services provided by wheat agro-ecosystems. The aim of this study was thus to quantify the effect of specific soil attributes (N cycling and soil physical properties), as affected by increased SOC concentration, on proxies for ecosystem services (food provision, water recharge, flood mitigation, filtering of N, and nitrous oxide regulation) in a diverse range of wheat cropping agro-ecosystems from around the world. Wheat agro-ecosystems were chosen as the focus of the study as wheat is an agricultural crop of global significance, with a large proportion of the world's population relying on wheat for their main source of nutrition and energy needs (FAO, 2015).

## Methods

### Overview

The Agricultural Production Systems sIMulator (APSIM; v.7.7; www.apsim.info; Holzworth et al., 2014) was used to simulate soil carbon, N, and water dynamics, as well as proxies for ecosystem services related to these dynamics, in a wheat cropping system at seven sites around the world. Simulations of wheat production in agro-ecosystems that had been previously parameterised and validated were used with slight modifications to make them applicable to this study. Proxies for ecosystem services that were likely to be influenced by N status and/or soil physical properties investigated in this study were selected from the available model outputs. At each site, simulations were undertaken at two SOC concentrations, one being the SOC concentration measured in the soils at the sites under long term agriculture and a second with higher SOC concentration reported in literature or estimated from simulations of management practices that aim to increase SOC. Four scenarios were simulated to isolate the relative effect of SOC [(a) measured SOC and (b) increased SOC] on soil properties [(a) N cycling and (b) soil physical properties including soil water holding capacity, bulk density, and saturated hydraulic conductivity]. While N cycling is currently linked to SOC in APSIM v7.7, there is no dynamic link between SOC and soil physical properties in this version of the model. A quantitative framework was thus developed to relate the values of the main parameters affecting soil water in APSIM to SOC concentration.

### Site descriptions

The seven study sites covered a range of soil textures, SOC contents, water management regimes, and climates (Table 1). Six sites were dryland agro-ecosystems and one (New Delhi) was irrigated. Wheat crops had been grown at all sites, and there was sufficient information available for the sites to allow configuration and, where necessary, testing of the model. Further details about the sites can be found in the publications relevant to each site (Table 1). These sites were selected for use in this study as they represented a diverse range of wheat production agro-ecosystems and had publications either providing details on previous successful modelling or sufficient information to allow model parameterisation and testing.

**Table 1:** **Information and soil properties for the seven study sites**.

**Study site**	**Balcarce**	**Brigalow**	**Canterbury**	**Liebe**	**New Delhi**	**Pendleton**	**Wageningen**
Country	Argentina	Australia	New Zealand	Australia	India	United States of America	The Netherlands
Latitude	37.50° S	26.84° S	43.63° S	30.27° S	28.38° N	45.72° N	51.97° N
Longitude	58.30° W	150.85° E	172.48° E	116.66° E	77.12° E	118.63° W	5.63° E
Average growing season	June–December	May–September	May–January	May–October	November–April	October–July	October–July
Average annual rainfall^1^ (mm)	928	636	738	316	694	317	784
Average annual temperature^1^ (°C)	14	20	11	19	24	11	10
Soil type	Clay loam	Clay	Silty loam	Sand	Sandy loam	Silty loam	Silty clay loam
SOC content 0.0–0.3 m (Total %)	2.58	1.10	1.72	0.40	0.40	1.31	2.80
Total annual irrigation (mm)	0	0	0	0	383	0	0
References	Asseng et al., 2013	Godde et al., 2016	Francis and Knight, 1993	Godde et al., 2016	Asseng et al., 2013	Rasmussen et al., 1998a,b	Asseng et al., 2013

1 *Average rainfall and temperature are calculated for the simulation time frame (between 30 and 81 years)*.

### Ecosystem services and outcome proxies

This study focussed on five proxies for ecosystem services (yield, drainage, infiltration, loss of N via deep drainage, and nitrous oxide emissions) for which SOC effects on N status and/or soil water holding capacity could be quantified (Table 2). For the filtering of N and nitrous oxide regulation services, APSIM could not provide direct measures of the services so we used N loss via deep drainage (kg N ha^−1^) and nitrous oxide emissions (kg N ha^−1^): These are not measures of the services but measures of outputs from the system and here used as proxies for the services. In addition, to better understand the effect of increased SOC concentration on nitrous oxide emissions, we determined the number of days that soil water was above Drained Upper Limit (DUL; equivalent to field capacity) as high soil moisture content (below saturation) facilitates denitrification.

**Table 2:** **Ecosystem service definition, proxy, and APSIM output variable**.

**Ecosystem service**	**Definition**	**Proxy from APSIM variable**	**APSIM output**
Food provision	Crop yield	Yield	Yield
Water recharge	Amount of water which drains through the soil profile to recharge aquifers	Water drained below the root zone	Annual drainage
Flood mitigation	Ability of soils to store and release water—amount of water which penetrates the soil surface	Rainfall—runoff	Annual infiltration = annual rainfall − annual runoff
Filtering of N	Amount of N attenuated by the soil	N via deep drainage from the soil profile	Annual loss of N via deep drainage
Nitrous oxide regulation	Amount of nitrous oxide attenuated by the soil	Nitrous oxide emitted	Annual nitrous oxide emissions

### Modelling

#### Model description

APSIM is a deterministic, daily time-step modelling framework, capable of simulating plant, soil, climate, and management interactions (Holzworth et al., 2014). It includes modules for: soil N and carbon dynamics (SoilN, Probert et al., 1998); soil water dynamics (SoilWat, Probert et al., 1998); surface organic matter (SurfaceOM, Probert et al., 1998); and a range of crop modules (e.g., Wheat, Wang et al., 2003). All modules are one dimensional and driven by meteorological data.

APSIM dynamically simulates changes in SOC and the resultant effect on N cycling (including soil mineral N dynamics), which in turn influences N supply to crops and N losses via deep drainage or denitrification. Carbon inputs to soils affect carbon flows between the carbon pools in APSIM, which in turn affects the corresponding N flows that are calculated using the C:N ratio of the receiving N pool. This functionality is central to the SoilN module in APSIM (Holzworth et al., 2014).

APSIM (v7.7) does not currently have the inbuilt capacity to dynamically simulate the effect of SOC on soil physical properties such as DUL, Lower Limit (LL15), and bulk density. The simulations were therefore modified (Section Representing the Effect of SOC on Soil Physical Properties in APSIM) so that values of these parameters varied in response to changes in SOC.

#### General model parameterisation

**Figure 1:**
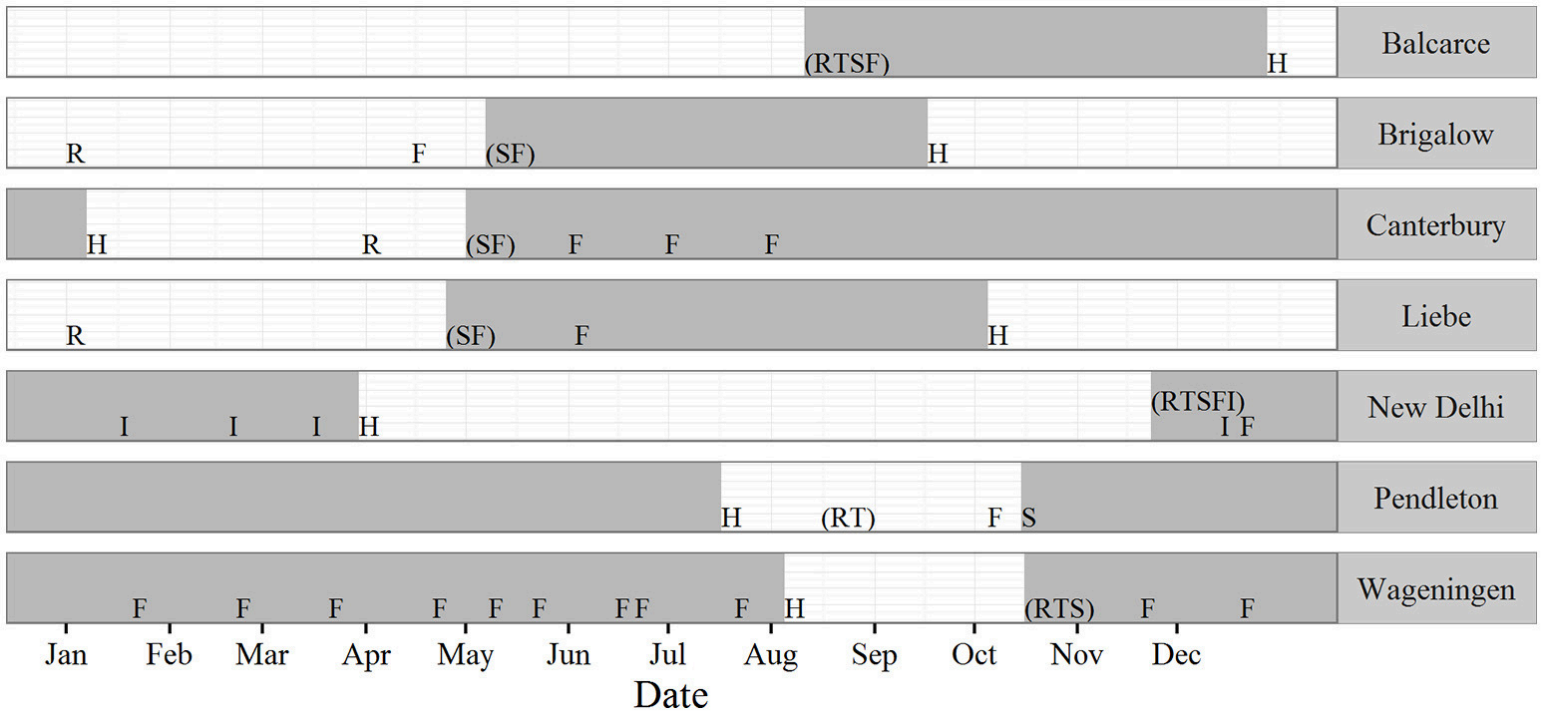
**Schedule of the main management operations simulated at each study site**. The shaded area represents the wheat crop growth period. Letters represent the following operations: R—reset of SOC, mineral N, water, and surface residue; T—tillage; S—Sowing date of wheat crop; F—fertiliser application; I—Irrigation; H—approximate harvest date of wheat crop. Letters within brackets indicate operations that occurred on the same day.

The Brigalow, Liebe, Wageningen, Balcarce, and New Delhi sites were parameterised with the soil and crop parameter values and management practices previously used to simulate these sites (Table 1, Tables S1, S2, and Figure 1). For consistency, for the Wageningen, Balcarce and New Delhi sites, the number of layers and layer thickness defined in the soil modules were adjusted from previous studies so there were three 0.1 m deep soil layers in the top 0.3 m of the soil. These changes to the soil layers had no effect on key output variables. The Pendleton and Canterbury sites were parametrised (Table 1, Tables S1, S2, and Figure 1) using published information (Table 1; Supplementary Material Section 4). Management operations were specified to reflect common practice in each region with a wheat cropping rotation followed by bare fallow simulated at all sites (Figure 1). To avoid long-term changes in soil model parameters during the simulations, SOC, mineral N, water, and surface residue values were reset annually to initial values. Reset dates were specified for each site to take into account the site and the agro-ecological conditions (Figure 1). Following the approach by Asseng et al. (2013), parameters for SOC, mineral N, water, and surface residue were reset to measured values of conditions at sowing for the Wageningen, Balcarce, and New Delhi sites. For the other sites measurements of conditions at sowing were not available. Thus, mineral N, water, and surface residue parameters were reset during the fallow with sufficient time to allow soil, water, and surface residue dynamics to establish prior to sowing. The date of annual output of non-yield parameters was 31st December.

The simulation time frame depended on the availability of reliable climate data (Table 3). For the Brigalow and Liebe sites, daily climate data were obtained from the Australian Bureau of Meteorology (via the SILO database, https://www.longpaddock.qld.gov.au/silo/; Jeffrey et al., 2001) for the meteorological stations nearest to the sites. On-site measurements of climate data were available for the Canterbury and Pendleton sites, with missing data in-filled with data from nearby meteorological stations. Climate data for the Balcarce, New Delhi, and Wageningen sites is described by Asseng et al. (2013).

**Table 3:** **Soil organic carbon, plant available water capacity and the simulation time frame for the seven study sites**.

**Variable**	**Balcarce**	**Brigalow**	**Canterbury**	**Liebe**	**New Delhi**	**Pendleton**	**Wageningen**
Measured SOC concentration 0.0–0.3 m (Total %)	2.6	1.1	1.7	0.4	0.4	1.3	2.8
Increased SOC concentration 0.0–0.3 m (Total %) simulated in *Nitrogen Cycling* and *Combined Properties* scenarios	3.3	1.8	2.6	1.1	2.0	2.6	4.3
Plant available water capacity (mm) in soil profile	238	221	183	136	121	246	354
Increased plant available water capacity (mm) in soil profile from effects of SOC on soil physical properties. Simulated in *Soil Physical Properties* and *Combined Properties* scenarios	241.0	223.5	190.3	145.0	137.7	254.0	360.0
Profile depth (m)	1.4	1.8	1.5	2.5	1.8	1.8	2.0
Simulation time frame	1981–2010	1963–2012	1972–2014	1963–2012	1980–2010	1930–2010	1980–2010

*See Tables S1, S2 for additional information about site specific parameter values*.

#### Scenarios

Four scenarios were simulated to determine the effect of N cycling and soil physical properties, as affected by increased SOC, on ecosystem service proxies (Table 4). In the *Control* scenario, the soil was simulated based on the measured SOC. In the second, the *Nitrogen Cycling* scenario, SOC was increased and that increase affected only N cycling. In this scenario the APSIM parameter for SOC was changed. In the third, the *Soil Physical Properties* scenario, increased SOC affected only soil physical properties and hence water supply to crops. In this scenario, the APSIM parameters bulk density, LL15, DUL, saturation, saturated hydraulic conductivity, and wheat lower limit were changed. To overcome the separation between SOC and soil physical properties in APSIM v7.7, we determined the effect that increased SOC would have on soil physical properties by using a method that was external to the model (Section Representing the Effect of SOC on Soil Physical Properties in APSIM) and modified the relevant parameters in the model to reflect the higher SOC. In the fourth, the *Combined Properties* scenario, SOC affected both N cycling and soil physical properties. Each scenario was simulated with seven N fertiliser rates (0, 50, 100, 150, 200, 250, 300 kg N ha^−1^). While some levels of N fertiliser would not be sensible to apply in particular wheat agro-ecosystems, these seven N fertiliser rates were simulated across all sites to understand the full response of the scenarios to N fertiliser applications.

**Table 4:** **The four scenarios simulated, identifying the level of SOC concentration affecting the soil properties**.

**Scenario**	**SOC concentration affecting N cycling**	**SOC concentration affecting soil physical properties**
Control	Measured SOC	Measured SOC
Nitrogen cycling	Increased SOC	Measured SOC
Soil physical properties	Measured SOC	Increased SOC
Combined properties	Increased SOC	Increased SOC

At each site, simulations were undertaken using the measured SOC (0.0–0.3 m soil depth) concentrations in the soils, and with site-specific higher SOC concentrations reported in literature or estimated from simulations of management practices that aim to increase SOC (Table 3). This approach to obtaining estimates of higher SOC concentration was used, as opposed to being increased by an arbitrary amount, to account for the differing soil carbon sequestration and storage capacities of different agro-ecosystems due to variation in climate, soils and past management. For the Brigalow and Liebe sites the higher SOC values were based on a study of attainable SOC sequestration for grain agro-ecosystems (Luo et al., 2014). For the Canterbury site the higher SOC value was based on SOC accumulation in field studies of different long-term crop management regimes (Francis et al., 1992; Francis and Knight, 1993). For the other sites, the higher SOC values were based on results of long-term (1,000 years, using repeated cycling of the existing meteorological record) simulations of the sites with management designed to increase SOC (e.g., manure application or cropping intensification) following the approach of Luo et al. (2014). Details of these simulations are given in the Supplementary Material Section 3.

#### Representing the effect of SOC on soil physical properties in APSIM

In the SoilWat module in APSIM, the primary parameters governing soil water dynamics are the water contents at saturation, DUL, and LL15, and saturated hydraulic conductivity (Probert et al., 1998). Bulk density is also implicated because of its relationship with saturation (Dalgliesh and Foale, 1998). Thus, our aim was to develop a system of equations that made these parameters a function of SOC (Supplementary Material Section 2). We used two different general approaches to link the parameters to SOC. For DUL and LL15, we used published pedotransfer functions (PTF) that predicted these water contents from SOC (and other soil parameters in some cases). For bulk density, saturation and saturated hydraulic conductivity we used more mechanistic approaches.

There are numerous PTFs reported in the literature that link DUL and LL15 to SOC (Table S3). Each PTF reflects the location and number of soils upon which it was developed (Cichota et al., 2013). Selecting a single PTF, e.g., as has been done previously (e.g., Porter et al., 2010), risks having a model framework that is only relevant for the soils on which the PTF was developed. In an effort to provide a more generally applicable framework we used an “ensemble” of 12 PTFs to develop equations for making DUL and LL15 dependant on SOC. Values of DUL and LL15 were predicted with each PTF over a range of SOC values and then fitted a function (which we term an ensemble PTF) across the range of SOC values (Supplementary Material Section 2.1) which reflects not the absolute DUL or LL15 but the change in DUL or LL15 for a unit change in SOC.

For bulk density, we used the approach of Adams (1973) which relates bulk density to the amount and density of soil organic matter in the soil (Supplementary Material Section 2.2). The water content at saturation was calculated from bulk density (Supplementary Material Section 2.3). To estimate saturated hydraulic conductivity, we used the semi-mechanistic function of Saxton and Rawls (2006) that relates saturated hydraulic conductivity to (1) water held at low suctions within larger pores that most effectively conduct water and (2) the slope of the soil moisture characteristic (Supplementary Material Section 2.4). This function is thus based on the water contents at saturation, DUL and LL15 and saturated hydraulic conductivity, and was calculated from these water contents at a given SOC value.

These parameters were modified in the *Soil Physical Properties* and *Combined Properties* scenarios. In these scenarios, the plant available water capacity in the top 0.3 m increased by between 2.5 and 16.7 mm, depending on the level of SOC increase and the soil texture at the site (Table 3).

### Statistical analysis

An analysis of variance was undertaken with the RStudio statistical package (v0.99.465) to test the simulated ecosystem services proxies for a significant difference between group (four scenarios and seven N fertiliser levels) means within a given site. Simulation years were used as replication in the analysis. If required, data were log transformed to meet the assumptions of normality and homogeneity of variance. The *post-hoc* Tukey's honest significant difference (HSD) test was used to analyse the ecosystem services proxies for a significant difference (*p* < 0.05) between the means for all scenario comparisons for a given fertiliser rate.

## Results

### Food provision ecosystem service quantified using yield as a proxy

**Figure 2:**
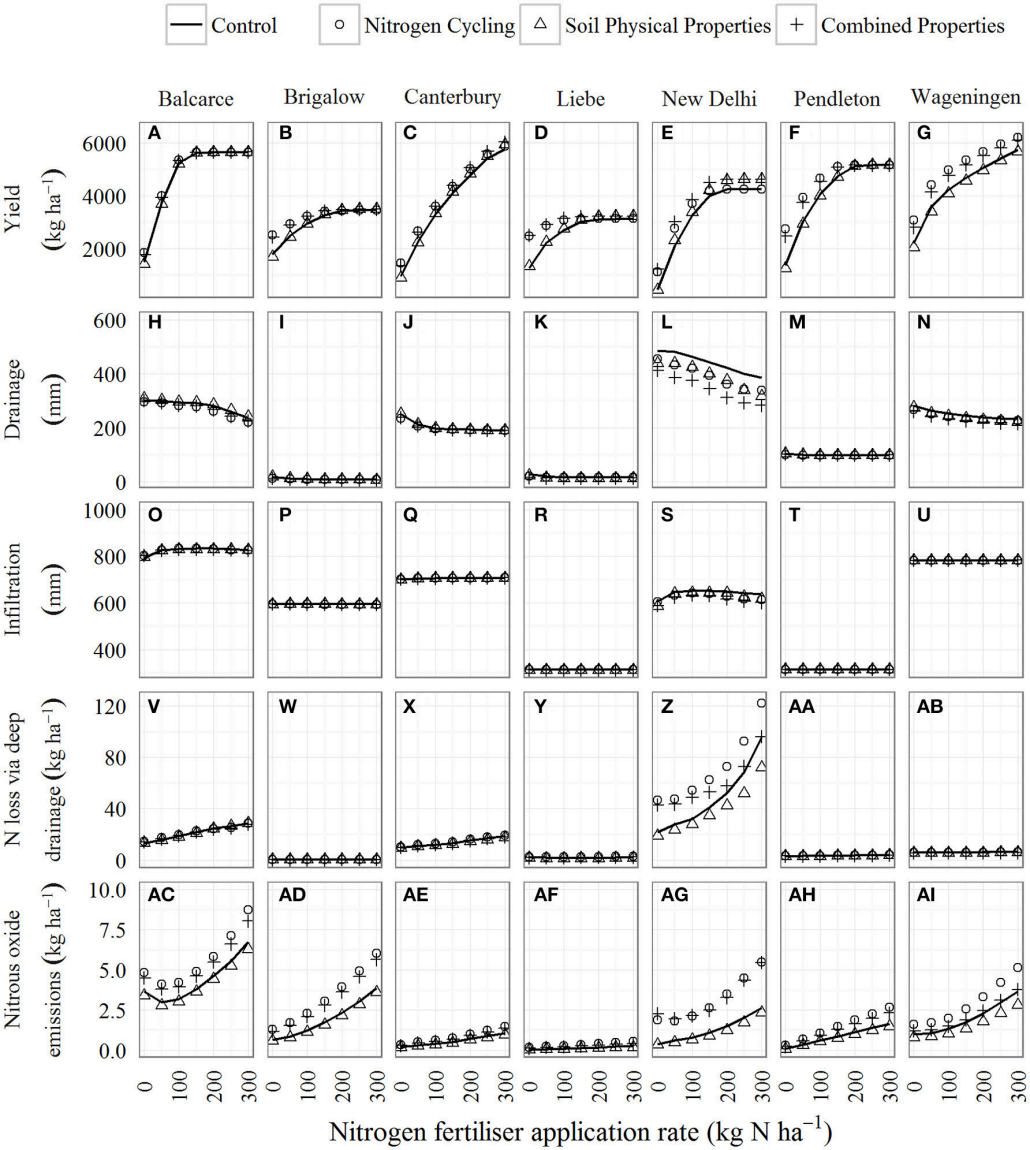
**Mean simulated values of five ecosystem service proxies for the ***Control*** (solid line) and the ***Nitrogen Cycling*** (open circle), ***Soil Physical Properties*** (open triangle), and ***Combined Properties*** (cross) scenarios, given increased soil organic carbon, for seven sites and seven nitrogen fertiliser rates from 0 to 300 kg N ha^**−1**^**. The data displayed represents ecosystem service proxies that have been simulated for between 30 and 80 years depending on the site. **(A–G)** display yield, **(H–N)** display drainage, **(O–U)** display infiltration, **(V–AB)** display N loss via deep drainage, and **(AC–AI)** display nitrous oxide emissions for the Balcarce, Brigalow, Canterbury, Liebe, New Delhi, Pendleton, Wageningen sites.

**Figure 3:**
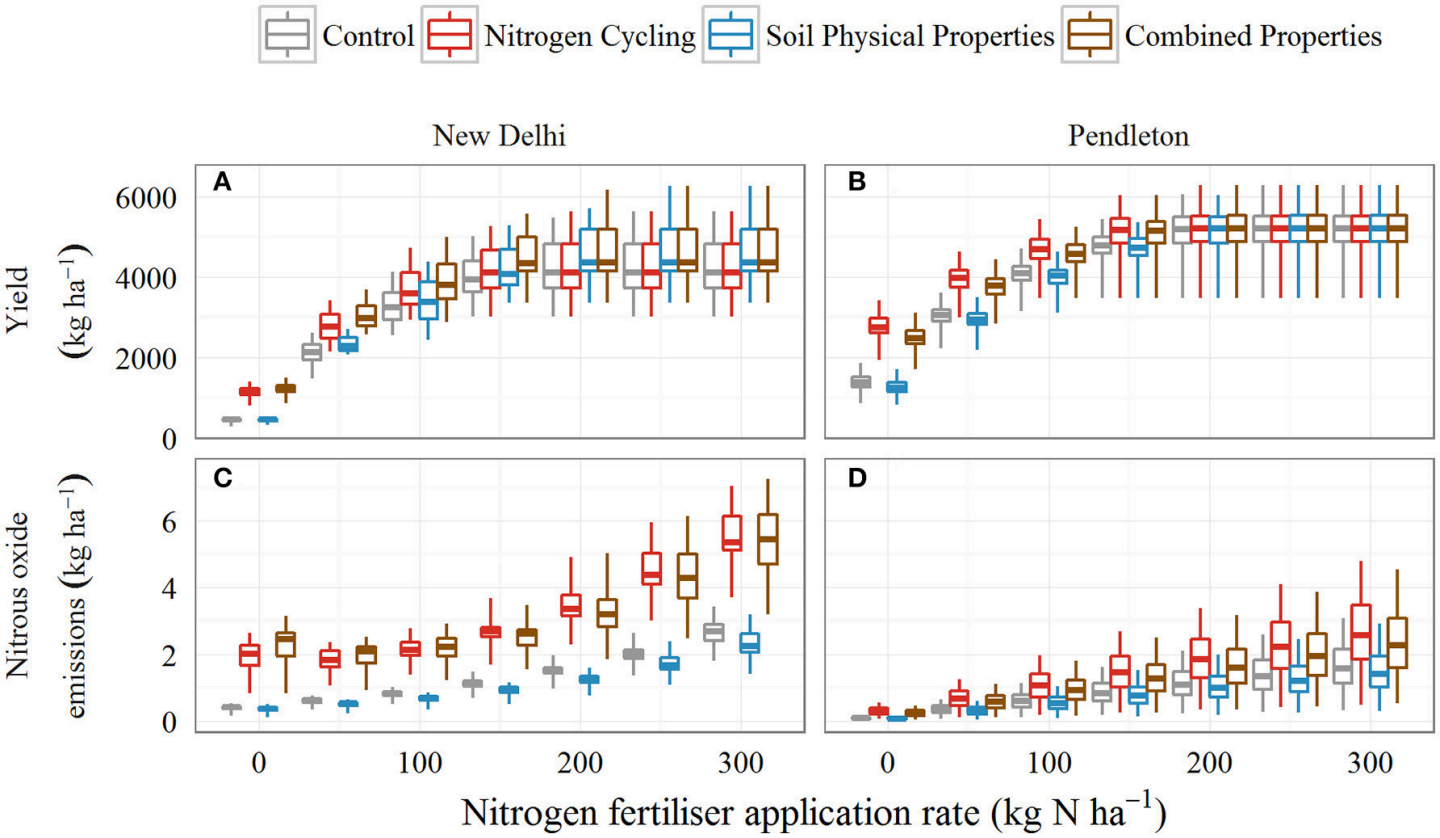
**A boxplot of simulated yield and nitrous oxide emissions for the ***Control*** and the ***Nitrogen Cycling, Soil Physical Properties***, and ***Combined Properties*** scenarios, given increased soil organic carbon, for the New Delhi (irrigated) and Pendleton (dryland) sites and seven nitrogen fertiliser rates from 0 to 300 kg N ha^**−1**^**. The data displayed represents ecosystem service proxies that have been simulated for 30 and 80 years, respectively. Boxes display the 25th and 75th quantile, the line in the box indicates the median and the whiskers extend from the minimum data value to the maximum. **(A,B)** display yield and **(C,D)** display nitrous oxide emissions for the Balcarce, Brigalow, Canterbury, Liebe, New Delhi, Pendleton, Wageningen sites.

Simulated mean wheat yield for the *Control* scenario varied between 464 and 5,783 kg ha^−1^ depending on site and N fertiliser rate (Figures 2A–G). At all sites, there was a significant (*p* < 0.05) effect of N fertiliser rate and of scenario, except at the Balcarce site where the effect of scenario was significant at *p* = 0.06. There was a significant interaction between scenario and N fertiliser rate at the New Delhi, Pendleton and Liebe sites (*p* < 0.05). Examples for the range of yield for the New Delhi and Pendleton sites are shown in (Figures 3A,B). The response of yield to the scenarios and N fertiliser rates for the Pendleton site is generally representative of the response for the other dryland sites.

In the *Nitrogen Cycling* scenario, higher SOC concentration significantly increased simulated wheat yields at low N fertiliser rates (i.e., 0 and 50 kg N ha^−1^) at all sites (Figures 2A–G). However, with the exception of the Wageningen site (Figure 2G), at high N fertiliser rates (i.e., 200, 250, and 300 kg N ha^−1^), higher SOC concentration had no significant effect on yields at any site (Figures 2A–G). For example, for the Pendleton site simulation with 0 kg N ha^−1^, the mean yield in the *Nitrogen Cycling* scenario was 1,374 kg ha^−1^ higher than the mean yield in the *Control* scenario (1,391 kg ha^−1^), whereas with 300 kg N ha^−1^ there was no difference in simulated yield (Figure 2F).

In the *Soil Physical Properties* scenario, the effect on simulated yields was much smaller than for the *Nitrogen Cycling* scenarios (Figures 2A–G). At low N fertiliser rates (i.e., 0 and 50 kg N ha^−1^), the yields in the *Soil Physical Properties* scenarios were generally either similar to or lower (although not significantly) than those in the *Control* scenarios. The exceptions to this were the Pendleton site, where yields were significantly lower than the *Control* at 0 kg N ha^−1^ (Figure 3B), and the New Delhi site, where yields were significantly higher at 50 kg N ha^−1^ (Figure 3A). At high N fertiliser rates (i.e., 200, 250, and 300 kg N ha^−1^), yields were slightly higher (although not significantly) than the *Control* at all sites except Wageningen. For example, for the Brigalow site with 0 kg N ha^−1^, the mean yield in the *Nitrogen Cycling* scenario was 95 kg ha^−1^ lower than the mean yield in the *Control* scenario, whereas with 300 kg N ha^−1^, it was 40 kg ha^−1^ higher (Figure 2B).

In the *Combined Properties* scenario, at low N fertiliser rates (i.e., 0 and 50 kg N ha^−1^) the effect of higher SOC concentration significantly increased simulated wheat yields at all sites to a similar degree as for the *Nitrogen Cycling* scenario (Figures 2A–G). The exception to this was the Brigalow site with 50 kg N ha^−1^ where there was no significant effect of the scenario. However, at high N fertiliser rates, simulated yields were higher (although not significantly) than the *Control* at all sites. The magnitude of the increases was similar to those in the *Soil Physical Properties* scenario, except for at the Wageningen site.

### Water recharge ecosystem service quantified using drainage as a proxy

Simulated mean drainage for the *Control* scenario was between 9 and 485 mm yr^−1^ depending on site and N fertiliser rate (Figures 2H–N). For all scenarios, drainage was lower for the dryland sites (mean drainage was between 8 and 309 mm yr^−1^) than for the irrigated New Delhi site (mean drainage was between 283 and 485 mm yr^−1^). There was a significant effect (*p* < 0.05) of N fertiliser rate on drainage at the Balcarce, Canterbury, New Delhi, and Wageningen sites, but not the Pendleton site (mean drainage at the Brigalow and Liebe sites was very small; between 8 and 30 mm yr^−1^. It is not considered further here).

While increased SOC concentration tended to decrease drainage for most sites, this was not significantly different from the *Control* for any site, N fertiliser rate, and scenario combination (Figures 2H–N). Simulated mean drainage was between 0.03 and 60 mm yr^−1^ lower than the *Control* for the *Nitrogen Cycling* scenario, between 7 mm higher and 69 mm lower for the *Soil Physical Properties* scenario, and 1 mm higher and 111 mm yr^−1^ lower for the *Combined Properties* scenario (Figures 2H–N). For the *Combined Properties* scenario, the largest decrease in drainage occurred at the irrigated New Delhi site where mean drainage was between 73 and 111 mm yr^−1^ lower than the *Control* (Figure 2L) whereas for the dryland sites, the greatest decrease in drainage was between 17 and 22 mm yr^−1^ lower than the *Control* at the Wageningen site (Figure 2N).

### Flood mitigation ecosystem service quantified using infiltration as a proxy

Simulated mean infiltration for the *Control* scenario varied between 314 and 833 mm yr^−1^ depending on site and N fertiliser rate (Figures 2O–U). There was no significant effect of N fertiliser rate or scenario on annual infiltration at any site. Depending on site, scenario, and N fertiliser rate, mean infiltration was between 9 mm yr^−1^ higher and 35 mm yr^−1^ lower than the *Control* (Figures 2O–U). Increased SOC had the greatest effect on infiltration at the New Delhi site, which was an irrigated site, where mean infiltration was between 3 and 35 mm yr^−1^ lower than the *Control* (Figure 2S).

### Filtering of N ecosystem service quantified using loss of N via deep drainage as a proxy

Simulated mean nitrate losses via deep drainage for the *Control* scenario varied between 0.2 and 96 kg ha^−1^ yr^−1^ depending on site and N fertiliser rate (Figures 2V–AB). There was a significant effect of N fertiliser rate on annual nitrate losses via deep drainage (*p* < 0.05) at the Balcarce, Canterbury, New Delhi, and Pendleton sites (Figures 2V,X,Z,AA). Simulated mean nitrate losses via deep drainage was between 1 kg ha^−1^ yr^−1^ lower and 27 kg ha^−1^ yr^−1^ higher than the *Control* for the *Nitrogen Cycling* scenario, between 0.1 kg ha^−1^ yr^−1^ higher and 24 kg ha^−1^ yr^−1^ lower for the *Soil Physical Properties* scenario, and between 2 kg ha^−1^ yr^−1^ lower and 21 kg ha^−1^ yr^−1^ higher for the *Combined Properties* scenario (Figures 2V–AB). While there was a varied effect of scenarios on annual nitrate losses via deep drainage, this was only statistically significant for the New Delhi site (Figure 2Z).

For the New Delhi site, the effect of higher SOC concentration on the *Nitrogen Cycling* scenario, significantly increased nitrate losses via deep drainage for lower N fertiliser rates of 0, 50, 100 kg N ha^−1^ (Figure 2Z). For the *Soil Physical Properties* scenario, higher SOC concentration decreased (although not significantly) annual nitrate losses via deep drainage for all N fertiliser rates. For the *Combined Properties* scenario, higher SOC concentration significantly increased annual nitrate losses via deep drainage for N fertiliser rates of 0 and 50 kg N ha^−1^.

### Nitrous oxide regulation ecosystem service quantified using nitrous oxide emissions as a proxy

Simulated mean nitrous oxide emissions for the *Control* scenario varied between 0.04 and 6.7 kg N_2_O-N ha^−1^ yr^−1^ depending on site and N fertiliser rate (Figures 2AC–AI). At all sites, there was a significant effect of N fertiliser rate and scenario (*p* < 0.05) on nitrous oxide emissions. Increased N fertiliser rate tended to increase nitrous oxide emissions, with the exception of the Balcarce and New Delhi sites where nitrous oxide emissions were lower for the 50 kg N ha^−1^ fertiliser rate than for 0 kg N ha^−1^. This counter-intuitive result can occur when low productivity from N stress for the 0 kg N ha^−1^ fertiliser rate scenarios can leave more N in the soil that is available for environmental loss than the 50 kg N ha^−1^ fertiliser rate scenarios, which yield higher (thus using a greater amount of soil N). At the Liebe, New Delhi and Pendleton sites, there was an interaction between scenario and N fertiliser rate (*p* < 0.05). Examples for the range of nitrous oxide emissions for the New Delhi and Pendleton sites are shown in (Figures 3C,D).

In the *Nitrogen Cycling* scenario, simulated mean annual nitrous oxide emissions were between 0.1 and 2.8 N_2_O-N kg ha^−1^ yr^−1^ higher than the *Control*, depending on site and N fertiliser rate (Figures 2AC–AI). The effect of higher SOC concentration significantly increased simulated nitrous oxide emissions across all N fertiliser rates at all sites. The exceptions to this were the Wageningen site where nitrous oxide emissions were significantly higher only at the 0 kg N ha^−1^ fertiliser rate and the Brigalow site where nitrous oxide emissions were only significantly higher at N fertiliser rates between 0 and 200 kg N ha^−1^.

In the *Soil Physical Properties* scenario, simulated mean nitrous oxide emissions were between 0.01 and 0.8 kg N_2_O-N ha^−1^ yr^−1^ lower than the *Control* (Figures 2AC–AI). However, Liebe and New Delhi were the only sites where nitrous oxide emission was significantly affected by this scenario. While the simulated nitrous oxide emissions were significantly lower than the control for the Liebe site, the values were extremely small (mean emissions were between 0.04 and 0.20 kg N_2_O-N ha^−1^ yr^−1^) and are not considered further here. For the New Delhi site, simulated nitrous oxide emissions were significantly lower than the *Control* for fertiliser rates between 100 and 300 kg N ha^−1^ (Figure 3C).

For the *Combined Properties* scenario, simulated mean nitrous oxide emissions were between 0.1 and 2.8 kg N_2_O-N ha^−1^ yr^−1^ higher than the *Control* (Figures 2AC–AI). Nitrous oxide emissions were significantly higher across all N fertiliser rates at the Canterbury, Liebe, New Delhi, and Pendleton sites (Figures 2AE,AF,AG,AH). The exception was Liebe site with 250 and 300 kg N ha^−1^ fertiliser where there was no significant effect of this scenario. For the Balcarce site, nitrous oxide emissions were significantly higher for N fertiliser rates of 150 kg N ha^−1^ and below (Figure 2AC). For the Brigalow site, nitrous oxide emissions were significantly higher for N fertiliser rates of 100 kg N ha^−1^ and below (Figure 2AD).

For the *Control* scenario, the mean number of days that soil water exceeded DUL was between 7 and 149 days yr^−1^, depending on site (Table 5). Scenarios reduced the days that soil water exceeded the DUL by between 0 and 16 days yr^−1^, depending on scenario and site.

**Table 5:** **Average number of days per year that soil water exceeded DUL for the ***Control, Nitrogen Cycling, Soil Physical Properties***, and ***Combined Properties*** scenarios**.

	**Mean number of days year^−1^ soil water exceeded DUL**	**Difference between the** ***Control*** **scenario for the mean number of days year**^**−1**^ **soil water exceeded DUL**		
**Site**	**Control**	**Nitrogen cycling**	**Soil physical properties**	**Combined properties**
Balcarce	149	−7	−2	−9
Brigalow	7	−1	0	−1
Canterbury	51	−1	−1	−2
Liebe	9	−1	−2	−3
New Delhi	96	−4	−12	−16
Pendleton	91	−1	−3	−4
Wageningen	134	−5	−6	−11

*Averages are for simulations run for between 30 and 80 years depending on the site for scenarios with seven N fertiliser rates*.

## Discussion

We disaggregated the effects of SOC on soil N cycling and soil physical properties to gain greater insights into the mechanisms underlying the effect of increased SOC concentration on ecosystem services. We found that increased SOC concentration in wheat production agro-ecosystems provided limited increase in ecosystem services. It is expected that an increased SOC will increase the N supply that contributes to grain yield and food provision (Aggarwal et al., 1997; Wani et al., 2003; Lal, 2006). Our results were consistent with that expectation, showing that with increased SOC concentration, N cycling was the major contributor to increased food provision (i.e., yields) when N was limiting (Figures 2A–G). However, when N was not limiting, as would often be likely for fertilised wheat production agro-ecosystems, N cycling from the increased SOC concentration provided no significant productivity benefit. This negligible effect of N cycling on yields at higher fertiliser rates was expected, as N fertiliser dominated the N supply at high fertiliser rates.

When N limited crop growth, i.e., at low N fertiliser rates, the N supply to the crop was dominated by N derived from mineralisation of organic N and our simulations showed the benefits of increased SOC concentration on crop production (Figures 2A–G). Importantly, N supply to crops from SOC is derived from the decomposition of soil organic matter, which can be considered “consumption” of the SOC natural capital as SOC stocks run down. In this study, SOC concentration was annually reset in simulations. This was done to avoid the confounding effects of long-term changes (run down) in SOC that would have eventuated in many of the wheat cropping systems simulated (e.g., at low rates of applied N fertiliser, in the absence of organic matter applications). An artefact of our methodology is that the average effect of SOC on crop N supply and production simulated in this study will be greater than generally seen in agro-ecosystems, where rundown would commonly occur (Dalal and Chan, 2001; Lal, 2004). The “consumption” of the SOC natural capital means that to derive the N-supply benefit in agro-ecosystems, SOC carbon levels benefit would need to be maintained through the use of management practices that increase SOC such as minimum tillage, stubble retention, and/or additions of organic matter (Paustian et al., 1992; Rasmussen and Parton, 1994; Luo et al., 2014; Smith et al., 2016). Even so, maintaining the high levels SOC concentration similar to those used in this study may be challenging in wheat agro-ecosystems because the SOC inputs from primary production may not be sufficient to sustain those SOC levels.

When all sites had the increased level of SOC concentration, we predicted that soil water holding capacity (0.0–0.3 m) would increase by <10 mm at six of the seven sites (Table 3) and 16 mm at the New Delhi site (Table 3). The findings from this study indicate that with increased SOC concentration, soil physical properties, and their effect of soil water dynamics caused no significant change to yield productivity [The exceptions to this were the Pendleton site, where yields for the *Soil Physical Properties* scenario were significantly lower than the *Control* at 0 kg N ha^−1^ (Figure 3B), and the New Delhi site, where yields were significantly higher at 50 kg N ha^−1^; Figure 3A] These findings contrast the widely held view that increased plant available water capacity from SOC increases productivity of farming systems (Duiker and Lal, 1999; Díaz-Zorita et al., 1999; Lal, 2006; Zhu et al., 2010; Hoyle et al., 2011; Barton et al., 2016; Williams et al., 2016). Many of these studies suggest (rather than provide empirical evidence) that increased water holding capacity from increased SOC plays a role in increasing productivity (Duiker and Lal, 1999; Zhu et al., 2010; Hoyle et al., 2011; Barton et al., 2016). However, some studies do provide evidence in support of this view. Williams et al. (2016) showed that increased water holding capacity from increased SOC reduced temporal variability of maize yields. In addition, Díaz-Zorita et al. (1999) found wheat yields were positivity correlated with soil water retention in dry years. The contrasting conclusions between this study and those found by Díaz-Zorita et al. (1999) and Williams et al. (2016) may reflect differences in scale or experiment design of the studies, and highlights the importance of multi-regional studies. Furthermore, it may be that the change in plant available water in this study was too small to show the benefits demonstrated in other studies. Further research is required to better understand the contribution of increased plant available water from increased SOC to crop productivity.

The increased level of SOC concentration increased the environmental footprint of agro-ecosystems. With increased SOC concentration, N cycling was the major contributor to nitrous oxide emissions (Figures 2AC–AI). Our results are consistent with other studies that have found nitrous oxide emissions increase with increased SOC (Li et al., 2005; Qiu et al., 2009; Ciais et al., 2010). While the increase in nitrous oxide emissions was relatively small, nitrous oxide is a potent greenhouse gas, and the extent of broadacre dryland cropping agro-ecosystems means small increases per unit area can lead to a considerable increase in nitrous oxide emissions for the agricultural sector globally. Conversely, there was no significant effect of soil physical properties, as affected by increased SOC concentration, on nitrous oxide emissions. High soil water contents contribute to increased denitrification, the process responsible for a large proportion of nitrous oxide emissions from soils (Weier et al., 1993; Smith et al., 1998). While increased SOC concentration increased soil water holding capacity compared with the *Control* (Table 3) it decreased the number of days that soil water content exceeded the threshold for denitrification to occur in the simulations (Table 5). Thus, the increased nitrous oxide emissions simulated with increased SOC resulted from the effect of SOC on N cycling.

There was no significant effect of increased SOC on the N losses via deep drainage from the dryland sites. Mean annual drainage was up to 30 times lower at the dryland sites compared with the New Delhi irrigated site (Figures 2H–N). At the New Delhi site, N cycling was the major contributor to increased loss of nitrate via deep drainage (Figure 2Z). Conversely, there was no significant effect of soil physical properties on loss of nitrate via deep drainage at this site. This supports studies that found high loss of N via deep drainage is associated with N inputs (Di and Cameron, 2002). The quantity of nitrate losses via deep drainage (filtering of N ecosystem service) is intimately linked with the quantity of water drained from the soil profile (water recharge ecosystem service). This highlights the links between services such as water recharge and filtering of N. On the one hand, water recharge is a desirable ecosystem service, but on the other hand, it may provide the catalyst for N to be lost via deep drainage, which can result in an increased environmental footprint for an agro-ecosystem.

While this study considered the effect of increased SOC on N cycling and soil physical properties, there are other soil processes such as cation exchange capacity and biological functions that are influenced by SOC. Further research could quantify the effect of SOC on processes such as structural stability, cation exchange capacity, and biological processes. Our study focussed on wheat agro-ecosystems, and the increases in SOC that might be achievable in those cropping systems. In other agricultural systems, larger increases in soil carbon are achievable. For example, in grazed pastoral systems considerable increases in SOC (Mg SOC ha^−1^) are possible in a relatively short time (Soussana et al., 2010; Machmuller et al., 2015) and is likely to have greater impacts on ecosystem service provision. Furthermore, climate change is likely to impact on the provision of ecosystem services from soils (Orwin et al., 2015). A similar approach to the one taken in this study could be used to investigate the effects of SOC on a wider range of agro-ecosystems under different climate change scenarios: such a study may enrich our understanding of the effects of SOC on ecosystem service provision.

The results of this study indicate that few of the ecosystem services provided by wheat agro-ecosystems are likely to be significantly affected by increased SOC. Furthermore, this study found that both the benefits and disadvantages to agro-ecosystems are likely to flow from the effect of SOC on N cycling, rather than from any effects of SOC on soil physical properties. Food provision may be significantly increased by increased SOC when N supply is limiting, which highlights that the ratio of contributions between natural and added fertility to total yield is a major sustainability indicator. However, when N supply is not limiting, no significant benefit will likely be derived. Increasing SOC is also likely to produce outcomes that will increase the environmental footprint of agro-ecosystems via two processes identified in this study. (a) Increased SOC led to significantly higher nitrous oxide emissions (nitrous oxide regulation ecosystem service) due the effect of SOC on N cycling, although the extent of increase was relatively small. (b) Higher N losses via deep drainage (filtering of N ecosystem service) at low N fertiliser rates in irrigated agro-ecosystems. Our results showed little change in annual infiltration or drainage and therefore the flood mitigation and water recharge ecosystem services are unlikely to be affected by increased SOC in dryland and irrigated wheat production agro-ecosystems.

## Author contributions

JP, PT, MP, NH, VS, JL, and WP conceived and designed the study. JP, JB, EM, ED, and MD conducted the simulations and analysed the data. JP, PT, ED, JB, MP, EM, and MD structured and wrote the manuscript. JP, PT, JB, ED, MP, EM, NH, MD, VS, JL, and WP edited the manuscript.

## Funding

The authors gratefully acknowledge financial support from the Australian Department of Agriculture and Water Resources, the Grains Research and Development Corporation, and the New Zealand Government to support the objectives of the Livestock Research Group of the Global Research Alliance on Agricultural Greenhouse Gases.

### Conflict of interest statement

The authors declare that the research was conducted in the absence of any commercial or financial relationships that could be construed as a potential conflict of interest.
