# Supplementary material for: Nitrogen Cycling from Increased Soil Organic Carbon Contributes Both Positively and Negatively to Ecosystem Services in Wheat Agro-Ecosystems
Source: Front Plant Sci. 2017 May 10;8:731. doi: 10.3389/fpls.2017.00731 (PMC5424304; doi:10.3389/fpls.2017.00731)
Supplement: Supplementary file 1 [file DataSheet1.docx]

Supplementary Material

Nitrogen Cycling from Increased Soil Organic Carbon Contributes Both Positively and Negatively to Ecosystem Services in Wheat Agro-Ecosystems

Jeda Palmer^*^, Peter J. Thorburn, Jody S. Biggs, Estelle J. Dominati, Merv E. Probert, Elizabeth A. Meier, Neil I. Huth, Mike Dodd, Val Snow, Joshua R. Larsen, William J. Parton

*** Correspondence:** Jeda Palmer: jeda.palmer@csiro.au; jeda.palmer@uqconnect.edu.au

# Model parameterisation

**Table S1.** Soil, water, and crop parameters used in APSIM for simulation of seven wheat production agro-ecosystems.

| **Variable** | **Balcarce** | **Brigalow** | **Canterbury** | **Liebe** | **New Delhi** | **Pendleton** | **Wageningen** |
| --- | --- | --- | --- | --- | --- | --- | --- |
| **SWCON**  **(0-1; 0.0 - 0.1 m)** | 0.5 | 0.3 | 0.8 | 0.7 | 0.5 | 0.5 | 0.5 |
| **Curve number**  **(0 - 100)** | 73 | 73 | 73 | 60 | 68 | 73 | 73 |
| **Initial water (mm) in soil profile** | 238 | 149 | 183 | 68 | 1 | 366 | 288 |
| **Initial soil nitrate and ammonium**  **(kg N ha^‑1^)** | 25 | 5 | 25 | 5 | 25 | 25 | 25 |
| **Initial surface residue (kg ha^-1^ wheat stubble)** | 1500 | 1000 | 1000 | 1000 | 1500 | 1500 | 1500 |
| **Cultivar** | Oasis | Hartog | Phoenix | Mace | HD 2009 | Pendleton dwarf^1^ | Arminda |
| **Rooting depth limit (m)** | 1.4 | 1.8 | 1.5 | 2.5 | 1.8 | 1.2 | 2.0 |

^1^Pendleton dwarf is a wheat variety parameterised for the Pendleton site according to long-term on-site measurements.

**Table S2.** Soil parameters used in APSIM for the *Control, Nitrogen Cycling, Soil Physical Properties* and *Combined Properties* scenarios in the top three soil layers (0.0-0.3 m) for the simulations of seven wheat production agro-ecosystems.

| **Site** | **Soil depth (m)** | **Soil organic carbon (Total %)** | | **LL15**  **(mm mm^-1^)^a^** | | **DUL (mm mm^-1^)** | | **Saturation**  **(mm mm^-1^)** | | **Bulk density**  **(Mg m^-3^)** | | **Saturated hydraulic conductivity (mm day^-1^)** | |
| --- | --- | --- | --- | --- | --- | --- | --- | --- | --- | --- | --- | --- | --- |
|  |  | Meas.  SOC | Incr. SOC | Meas.  SOC | Incr. SOC | Meas.  SOC | Incr. SOC | Meas.  SOC | Incr. SOC | Meas.  SOC | Incr. SOC | Meas.  SOC | Incr. SOC |
| **Balcarce** | 0.0-0.1 | 3.225 | 4.245 | 0.165 | 0.180 | 0.365 | 0.390 | 0.461 | 0.480 | 1.075 | 1.000 | 59 | 46 |
|  | 0.1-0.2 | 3.300 | 3.980 | 0.170 | 0.180 | 0.354 | 0.370 | 0.448 | 0.460 | 1.100 | 1.050 | 54 | 45 |
|  | 0.2-0.3 | 1.200 | 1.540 | 0.180 | 0.180 | 0.356 | 0.370 | 0.426 | 0.430 | 1.150 | 1.120 | 23 | 21 |
| **Brigalow** | 0.0-0.1 | 1.190 | 2.250 | 0.190 | 0.210 | 0.390 | 0.430 | 0.440 | 0.490 | 1.420 | 1.290 | 125 | 150 |
|  | 0.1-0.2 | 1.100 | 1.800 | 0.190 | 0.210 | 0.385 | 0.410 | 0.435 | 0.470 | 1.420 | 1.330 | 123 | 137 |
|  | 0.2-0.3 | 1.010 | 1.360 | 0.190 | 0.200 | 0.380 | 0.390 | 0.430 | 0.450 | 1.420 | 1.430 | 121 | 127 |
| **Canterbury** | 0.0-0.1 | 2.251 | 3.601 | 0.154 | 0.170 | 0.374 | 0.420 | 0.456 | 0.500 | 1.311 | 1.170 | 4832 | 5363 |
|  | 0.1-0.2 | 1.986 | 2.886 | 0.154 | 0.160 | 0.363 | 0.390 | 0.447 | 0.480 | 1.344 | 1.240 | 4809 | 5335 |
|  | 0.2-0.3 | 0.925 | 1.375 | 0.152 | 0.150 | 0.320 | 0.340 | 0.413 | 0.430 | 1.474 | 1.410 | 4715 | 5254 |
| **Liebe** | 0.0-0.1 | 0.670 | 1.659 | 0.040 | 0.050 | 0.100 | 0.150 | 0.370 | 0.420 | 1.600 | 1.450 | 1158 | 1339 |
|  | 0.1-0.2 | 0.290 | 0.949 | 0.050 | 0.050 | 0.110 | 0.140 | 0.360 | 0.400 | 1.640 | 1.530 | 900 | 1038 |
|  | 0.2-0.3 | 0.250 | 0.580 | 0.050 | 0.050 | 0.110 | 0.130 | 0.370 | 0.390 | 1.600 | 1.550 | 1006 | 1074 |
| **New Delhi** | 0.0-0.1 | 0.450 | 2.819 | 0.110 | 0.130 | 0.174 | 0.270 | 0.368 | 0.470 | 1.560 | 1.250 | 393 | 498 |
|  | 0.1-0.2 | 0.400 | 1.979 | 0.109 | 0.120 | 0.173 | 0.240 | 0.368 | 0.440 | 1.575 | 1.350 | 399 | 423 |
|  | 0.2-0.3 | 0.350 | 1.140 | 0.107 | 0.110 | 0.172 | 0.210 | 0.368 | 0.410 | 1.590 | 1.470 | 407 | 465 |
| **Pendleton** | 0.0-0.1 | 1.420 | 3.340 | 0.110 | 0.130 | 0.320 | 0.380 | 0.520 | 0.580 | 1.240 | 1.070 | 562 | 531 |
|  | 0.1-0.2 | 1.310 | 2.590 | 0.110 | 0.120 | 0.320 | 0.360 | 0.520 | 0.560 | 1.245 | 1.130 | 562 | 550 |
|  | 0.2-0.3 | 1.200 | 1.840 | 0.110 | 0.120 | 0.320 | 0.340 | 0.520 | 0.540 | 1.250 | 1.190 | 562 | 561 |
| **Wageningen** | 0.0-0.1 | 2.800 | 4.980 | 0.180 | 0.230 | 0.390 | 0.470 | 0.490 | 0.570 | 1.350 | 1.130 | 66 | 78 |
|  | 0.1-0.2 | 2.800 | 4.250 | 0.180 | 0.210 | 0.390 | 0.440 | 0.490 | 0.550 | 1.350 | 1.190 | 66 | 76 |
|  | 0.2-0.3 | 2.800 | 3.530 | 0.180 | 0.200 | 0.390 | 0.420 | 0.490 | 0.520 | 1.350 | 1.270 | 66 | 72 |

^a^ Crop lower limit was the same as soil LL15.

# Framework for making soil hydraulic properties dependent on soil carbon

In this section we describe the system of equations for making the primary parameters governing soil water dynamics in APSIM (Probert et al., 1998) a function of SOC. These parameters are the water contents at saturation (SAT), Drained Upper Limit (DUL) and Lower Limit (LL15), saturated hydraulic conductivity (KSat) and bulk density (BD). We used an empirical approach based on published pedotransfer functions (PTF) for DUL and LL15, and more mechanistic approaches for the other parameters.

## Drained upper limit and lower limit

Pedotransfer functions have been developed to predict the soil water content at either specific matric suctions (10, 33 and 1500 kPa) or any given suction. The relationship between matric suction and DUL is not precise. However, DUL might be defined as the water content at matric suction values of either 10 or 33 kPa. Lower Limit is commonly defined as the water content at a matric suction value of 1500 kPa. We selected 12 PTFs to estimate volumetric soil water content at matric suction values of 10 and/or 33 kPa and 1500 kPa (Figure S1). Many of these PTFs also require specification of soil particle size (e.g. clay and/or silt content) and BD. Some PTFs were developed on soil organic matter content rather than SOC. For these functions soil organic matter content was calculated from SOC using the conversion factor of 1.72 (Nelson and Sommers, 1982).

There is a range in water contents predicted by the different PTFs (Figure S1). This range is likely to come from the different types and numbers of soils included in the different studies (Wösten et al., 2001). To provide the most generally applicable relationships between water content and SOC, we treated the range of PTFs as an ensemble and developed a function (termed the ensemble PTF) to represent the mean water content across the different PTFs. This follows the similar approach used by Cichota et al. (2013). To develop the ensemble PTF, soil water content for matric suction values of 10 kPa, 33 kPa and 1500 kPa were calculated with each PTF for SOC values between 0.5 and 6 % at 0.1 % increments (e.g., 0.5%, 0.6%, 0.7%,..., 6%) (Figure S1). Then, a quadratic function fitted to the data, i.e.:

*x* = *a* + *b*.SOC + *c*.SOC^2^ (S1)

where, *x* is the water content at the different matric suction values, and a, b, and c are the equation coefficients. The resultant values of the coefficients *b* and *c* are given in Table S5.


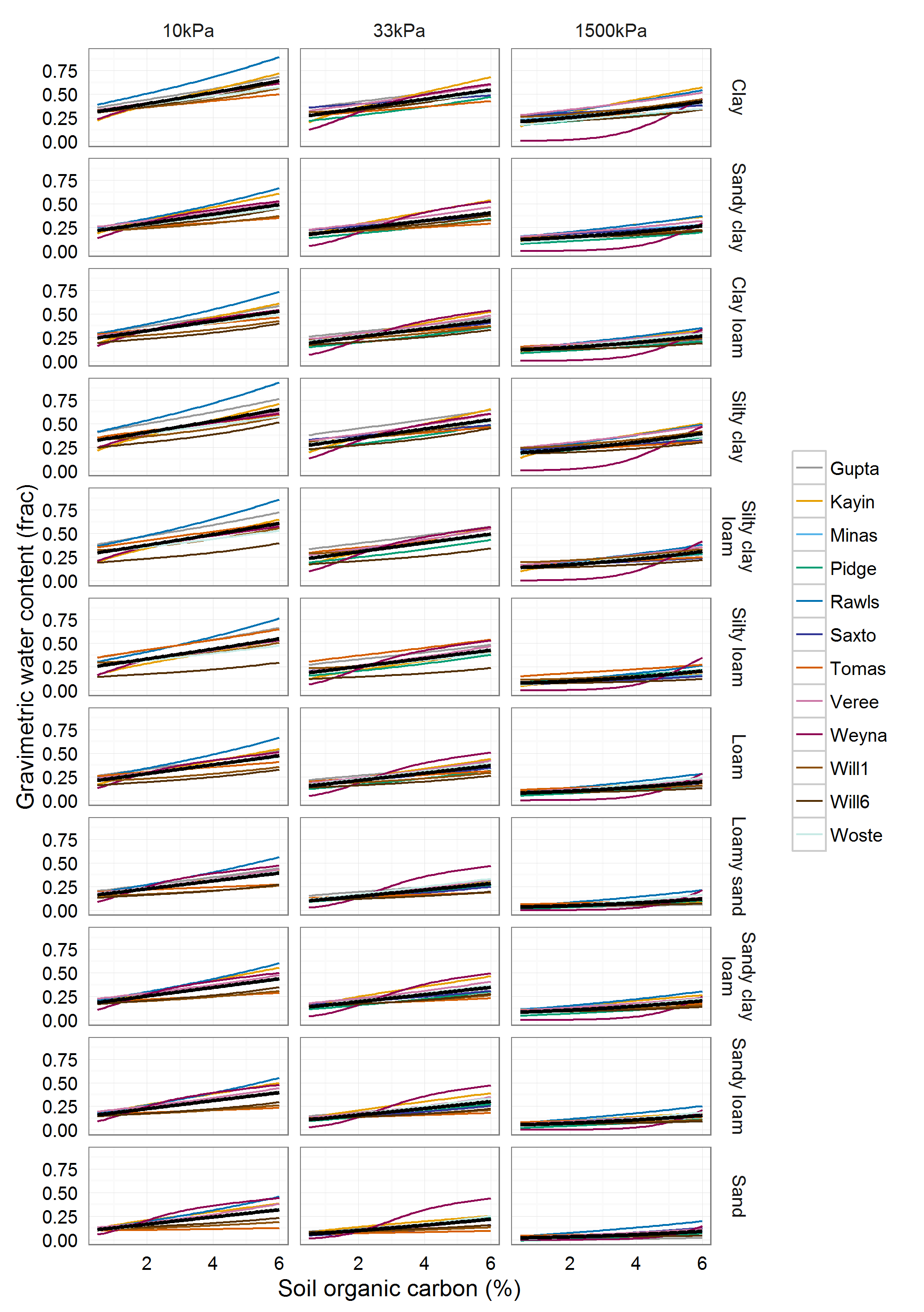


**Figure S1**. Gravimetric water content at matric suction values of 10, 33 and 1500 kPa for 11 soil texture classes estimated from published PTFs for soil organic carbon content ranging from 0.5 to 6 %. The black solid line represents the quadratic function fit to the data, or ensemble PTF. The legend displays the name of the first author who developed a given PTF, listed in Table S3, in abbreviated form.

The value of the coefficient *a* was **not** determined from fitting Equation S1 to the PTF data. For a given SOC concentration the different PTFs can substantially differ in their prediction of DUL or LL15 (e.g. Figure S1). A large amount of this variation is caused by the different intercepts (i.e. value of DUL or LL15 at low SOC concentrations) of the PTFs. Thus the relevance of a particular PTF, or the ensemble PTF will be increased if the value of *a* can be derived from knowledge of SOC, DUL and LL15 for a particular soil the soil being simulated. This approach is consistent with the philosophy promoted in application of the APSIM model, where users are encouraged to set values of the parameters SOC, DUL and LL15 (and most other soil parameters) from measured soil properties. Thus the value of *a* was calculated from:

*a* = *x*_t=0_ - *b*.SOC _t=0_ - *c*.SOC^2^ _t=0_ (S2)

where, the super script t=0 is the initial measured value.

All of the PTFs for DUL and LL15 required inputs in addition to SOC. Many require BD (as a function of SOC) as an input. There is a mechanistic function relating BD to SOC (Adams, 1973), but it requires data on BD and SOC to initialise coefficient values. That information is not available in the context of deriving Equation S1. Instead, we used published PTFs (Table S4). We used the arithmetic mean of the published PTFs to provide a more generally-applicable equation (i.e. an ensemble PTF) from the ensemble (Figure S2).


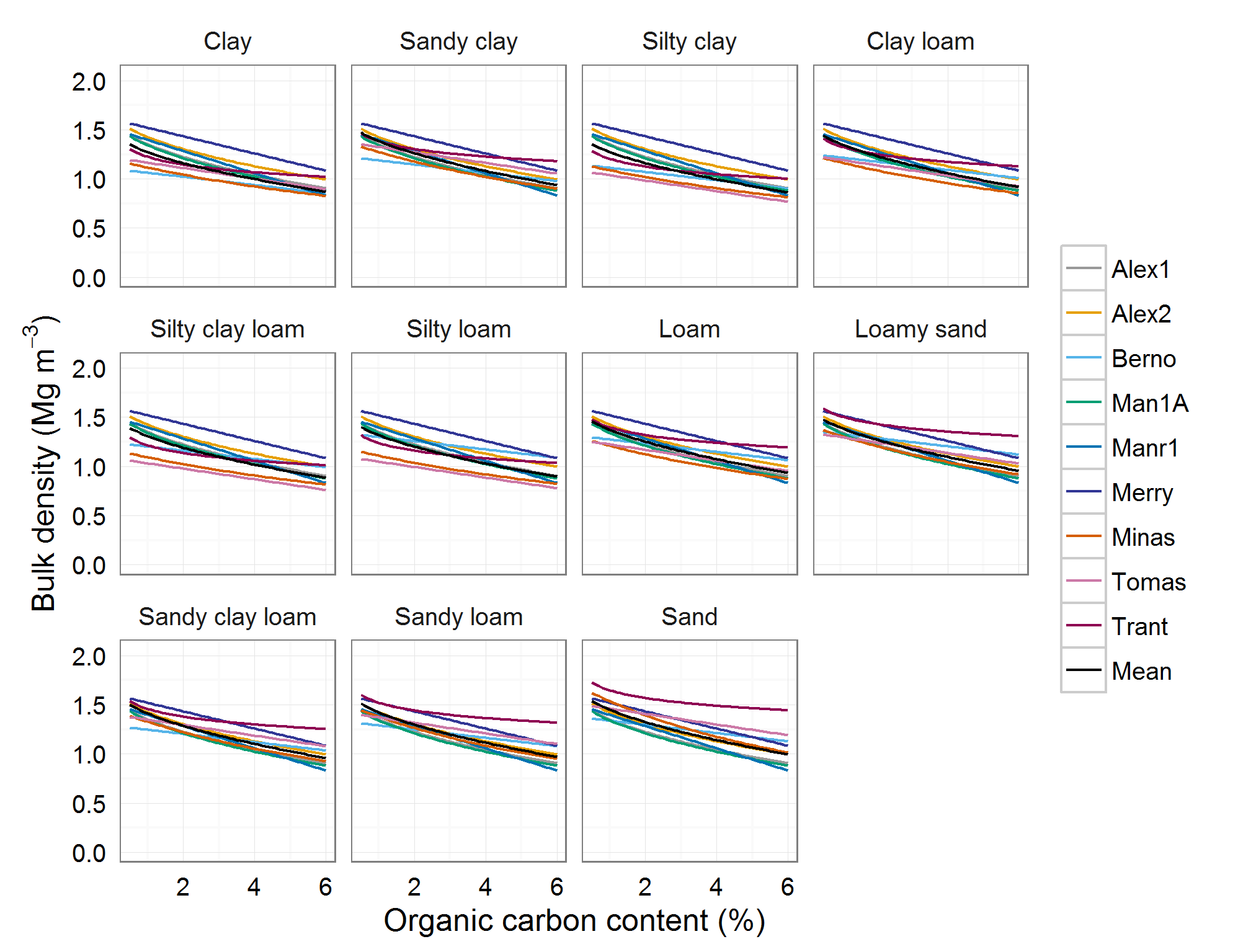


**Figure S2.** Bulk density for 11 soil texture classes estimated from published PTFs for organic carbon content ranging from 0.5 to 6 %. The black solid line represents the arithmetic mean of the data. The grey soil line shows the function developed by Adams (1973) using the mean value at 0.5 % SOC as the initial bulk density value. The legend displays the name of the first author who developed a given PTF, listed in Table S4 in abbreviated form.

Many of the PTFs for DUL and LL15 also require information on soil particle size (e.g. the proportion of clay and/or silt) as an input. The particle size boundary between clay and silt varied depending on the textural classification system used to describe the soils on which the PTF was developed; the boundary was 0.05 mm for PTFs developed using the USDA/FAO classification system (commonly used in the United States of America) and 0.02 mm for those developed using the International/Australian classification system (International Society of Soil Science 1929; commonly used in Australia). Where necessary, data were converted between the USDA/FOA and International size definition using the relationships developed by Minasny and McBratney (2001).

In the PTF developed by Wosten et al. (1999) soils were differentiated as “top-soil” or “sub-soil”. Wosten et al. (1999) defined top-soil and sub-soil as qualitative variables having the value of 1 or 0 respectively.

One PTF (Williams *et al*., 1992; Function 6) segregated soils based on their classification by Northcote (1971). Soil texture and other available profile attributes were used to assign soils to a Northcote class.

**Table S3.** Summary of published pedotransfer functions used to estimate water retention characteristics.

| **Reference** | **Reference code** | **Country** | **Pedotransfer function** |
| --- | --- | --- | --- |
| Gupta and Larson (1979) | Gupta | United States of America | θ _v,10_= (0.005018*P50_2000) + (0.008548*P2_50) + (0.008833*P2) + (0.004966*(OC*1.72)) - (0.2423*BD)  θ_v,33_ = (0.003075*P50_2000)+ (0.005886*P2_50) + (0.008039*P2) + (0.002208*(OC*1.72)) - (0.1434*BD)  θ _v,1500_= (-0.000059*P50_2000)+ (0.001142*P2_50) + (0.005766*P2) + (0.002228*(OC*1.72)) - (0.02671*BD) |
| Kay in (Krull et al. 2004) | Kayin | Canada | θ*_v_* = a\|(Ψ/1000)\|^b^  Where:  a = exp(-4.1518 + (0.6851*ln(P2)) + (0.4025*ln(OC)) + (0.2731*ln(BD)))  b = -0.5456 + (0.1127*ln(P2) + (0.0233*ln(OC)) + (0.1013*ln(BD))) |
| Rawls et al. (1992) | Rawls | United States of America | θ_v,10_ = 0.4118 - (0.003*P50_2000) + (0.0023*P2) + (0.0317*(OC*1.72)  θ_v,1500_ = 0.026 + (0.005*P2) + (0.0158*(OC*1.72)) |
| Saxton and Rawls (2006) | Saxto | United states of America | θ_v,33_ = -0.015 + 0.626*a + 1.283*a^2  θ_v,1500_ = -0.02 + 1.14*(0.031 - 0.024*P50_2000/100 + 0.487*P2/100 + 0.006*(OC*1.72) + 0.005*P50_2000/100*(OC*1.72) - 0.013*P2/100*(OC*1.72) + 0.068*P50_2000/100*P2/100)  Where:  a = 0.299 - (0.251*P50_2000/100) + (0.195*P2/100) + (0.011*(OC*1.72)) + (0.006*P2/100*(OC*1.72)) - (0.027*P2/100*(OC*1.72)) + (0.452*P50_2000/100*P2/100) |
| Tomasella et al. (2000) | Tomas | Brazil | θ (Ψ) = θ_R_ + (θ_S_ - θ_R_) [1 + \|α Ψ\|^n^]^m^  Where:  θ_S_ = (91.6203 - (30.0046*BD) + (1.5925*OC) + (0.0022*P50_2000C*P2_50) - (0.0036*P50_2000C*P2) - (0.0018*P50_2000C^2) - (0.001*P50_2000F^2))/100  θ_R_ = (23.3867 + (0.1103*P2) - (4.7949*BD) + (0.0047*P2_50*P2) - (0.0027*P50_2000C^2) - (0.0022*P50_2000F^2) - (0.0048*P2_50^2))/100  α = exp((205.6546 - (2.556*P2_50) - (0.1329*P2) - (247.4904*BD) + (0.0189*P50_2000C*P50_2000F) + (0.1177*P2_50*P50_2000C) + (0.0517*P50_2000F*P2) +  (0.0617*P50_2000C^2))/100)  *n* = (168.8617 - (0.0258*P50_2000C*P2_50) - (0.0261*P50_2000F*P2) + (0.0093*P50_2000F^2) - (0.0077*P2_50^2))/100  m = 1-1/*n* |
| Vereecken et al. (1989) | Veree | Belgium | θ (Ψ) = θ_R_ + (θ_S_ - θ_R_) [1 + \|α Ψ*10\|^n^]^m^  Where:  θ_S_ = 0.81 - (0.283*BD) + (0.001*P2)  θ_R_ = 0.015 + (0.005*P2) + (0.014*OC)  α = exp(-2.486 + (0.025*P50_2000) - (0.351*OC) - (2.617*BD) - (0.023*P2))  *n* = exp(0.053 - (0.009*P50_2000) - (0.013*P2) + (0.00015*(P50_2000^2)))  m = 1 |
| Weynants et al. (2009) | Weyna | Belgium | θ (Ψ) = θ_R_ + (θ_S_ - θ_R_) [1 + \|α Ψ*10\|^n^]^-m^  Where:  θ_S_ = 0.6355 + (0.0013*P2) - (0.1631*BD)  θ_R_ = 0  α = exp(-4.3003 - (0.0097*P2) + (0.0138*P50_2000) - (0.992*OC))  *n* = 1 + exp(-1.0846 - (0.0236*P2) - (0.0085*P50_2000) + (0.0001*P50_2000^2))  m = 1 |
| Williams et al. (1992) Function 1 | Will1 | Australia | θ_V_ = (exp(a + b In \|Ψ/100\|))/100  Where:  a = 2.57 + (0.238*ln(P2)) - (0.000192*(P20_2000F^2)) - (0.0137*P20_2000C) + (0.122*ln(P20_2000C)) - (0.0926*ln(OC*1.72)) + (0.0412*(OC*1.72))  b = -0.403 + (0.0871*ln(P2)) + (0.0014*P20_2000C) - (0.00077*P20_2000F) |
| Williams et al. (1992) Function 6 | Will2 | Australia | θ_V_ = (exp(a + b In\|Ψ/100\|))/100  Where:  a = 2.124 + (0.1257*(ln(P2^2))) - (0.00551*P20_2000F) + (0.0848*NorthcoteID) + (0.00238*(OC*1.72)^2) - (0.00398*P2_20)  b = -0.292 - (0.00128*P20_2000F) - (0.0189*ln(OC*1.72)) + (0.0282*ln(NorthcoteID)) + (0.0627*ln(P2)) - (0.00002*P2^2) |
| Wosten et al. (1999) | Woste | 12 European countries | θ (Ψ) = θ_R_ + (θ_S_ - θ_R_) [1 + \|α Ψ*10\|^n^]^-m^  Where:  θ_S_ = 0.7919 + (0.001691*P2) - (0.2961*BD) - (0.000001491*P2_50^2) + (0.0000821*(OC*1.72)^2) + (0.02427/P2) + (0.01113/P2_50) + (0.01472*log(P2_50)) -(0.0000733*P2*(OC*1.72)) - (0.000619*BD*P2) - (0.000001183*BD*(OC*1.72)) -(0.0001664* soilLayer*P2_50)  θ_R_ = 0  α = exp(-14.96 + (0.03135*P2) + (0.0351*P2_50) + (0.646*(OC*1.72)) + (15.29*BD) - (0.192*1) - (4.671*BD^2) - (0.000781*P2^2) - (0.00687*(OC*1.72)^2) + (0.0499/(OC*1.72)) + (0.0663*log(P2_50)) + (0.1482*log((OC*1.72))) - (0.04546*BD*P2_50) - (0.4852*BD*(OC*1.72)) + (0.00673* soilLayer*P2))  *n* = 1 + exp(-25.23 - (0.02195*P2) + (0.0074*P2_50) - (0.194*(OC*1.72)) + (45.5*BD) (7.24*BD^2) + (0.0003658*P2^2) + (0.002885*(OC*1.72)^2) - (12.81/BD) - (0.1524/P2_50) - (0.01958/(OC*1.72)) - (0.2876*log(P2_50)) - (0.0709*log((OC*1.72))) - (44.6*log(BD)) - (0.02264*BD*P2) + (0.0896*BD*(OC*1.72)) + (0.00718*soilLayer*P2))  m = 1-1/*n* |
| Minasny and Hartemink (2011) | Minas | Tropical countries | θ_v,1500_ = (7.95 + (0.86*OC) + (0.4*P2) - (0.004*(P2-37.7)^2))/100 |
| Pidgeon (1972) | Pidge | Uganda | θ_v,33_ = (7.38 + (0.16*P2_20) + (0.3*P2) + (1.5*(OC*1.72)))/100  θ_v,1500_ = ((-4.19 + (0.19*P2_20) + (0.39*P2) + (0.9*(OC*1.72))))/100 |

Where: θ_v_, volumetric water content; θ_v,10_, volumetric water content at a matric suction value of 10 kPa; θ_v,33_ volumetric water content at a matric suction value of 33 kPa; θ_v,1500­_, volumetric water content at a matric suction value of 1500 kPa; P2, soil particle size < 0.002 mm, P2_50, soil particle size 0.002 - 0.05 mm, P50_2000, soil particle size 0.05 - 2 mm; P2_20, soil particle size 0.002 - 0.02 mm, P20_2000, soil particle size 0.02 - 2 mm; P20_2000F, P20_2000*0.7; P20_2000C, P20_2000*0.3; P50_2000F, P50_2000*0.7; P50_2000C, P50_2000*0.3; OC, soil organic carbon %; BD, bulk density; Ψ, matric potential kPa; ln, natural logarithm; exp, exponential; NorthcoteID, texture class (1-6) according to Northcote (1971); soilLayer, qualitative variables referring to top-soil or sub-soil having the value of 1 or 0 respectively.

**Table S4.** Summary of pedotransfer functions used to estimate bulk density.

| **Reference** | **Reference code** | **Country** | **Pedotransfer function** |
| --- | --- | --- | --- |
| Alexander (1980) Function 1 (Upland soils) | Alex1 | United States of America | BD = 1.66 - 0.308*(OC^0.5)) |
| Alexander (1980) Function 2  (Alluvial soils) | Alex2 | United States of America | BD = 1.72 - 0.295*(OC^0.5)) |
| Bernoux et al. (1998) | Berno | Brazil | BD = 1.398 - (0.0047*P2) - (0.042*OC)) |
| Manrique and Jones (1991) Function 1 | Man1A | United states of America, Hawaii, Puerto Rico and others | BD = 1.51 - (0.113*OC)) |
| Manrique and Jones (1991) Function 1A | Manr1 | United states of America, Hawaii, Puerto Rico and others | BD = 1.66 - (0.318*OC^0.5)) |
| Merry in Spouncer et al. (2000) | Merry | Australia | BD = 1.608 - (0.0872*OC)) |
| Minasny and Hartemink (2011) | Minas | Tropical countries | BD = 100/(((OC*1.72)/BD_OM_) + ((100 - (OC*1.72))/ 0.935 + 0.049*ln(Depth) + 0.0055*P50_2000 + 0.000065*(P50_2000 - 38.96)^2))) |
| Tomasella and Hodnett (1998) | Tomas | Brazil | BD = 1.578 - (0.054*OC) - (0.006*P2_50) - (0.004*P2)) |
| Tranter et al. (2007) modified by Murphy (2015) | Trant | Australia | BD = (1.35 + (0.0045*P20_2000) + (0.00006*(44.7-P20_2000)^2) + (0.06 * ln(Depth))) – (0.217 + (0.114*ln(OC)) + (0.077*ln(Depth))) |

Where: BD, bulk density Mg m^-3^; OC, soil organic carbon %; P2, soil particle size < 0.002 mm, P2_50, soil particle size 0.002 - 0.05 mm, P50_2000, soil particle size 0.05 - 2 mm; P2_20, soil particle size 0.002 - 0.02 mm, P20_2000, soil particle size 0.02 - 2 mm; BD_OM_, organic matter bulk density = 0.224 Mg m^−3^; Depth, depth of soil layer cm; ln, natural logarithm.

**Table S5.** Coefficients for the ensemble PTF developed to estimate gravimetric water content at matric suction values of 10, 33 and 1500 kPa.

| **Soil class** | **Gravimetric water content** | | | | | | | | |
| --- | --- | --- | --- | --- | --- | --- | --- | --- | --- |
|  | **10 kPa** | | | **33 kPa** | | | **1500 kPa** | | |
|  | Constant a | b | c | Constant  a | b | c | Constant  a | b | c |
| **Clay** | 0.28781 | 0.05395 | 0.00082 | 0.24808 | 0.04942 | 0.00001 | 0.19874 | 0.02100 | 0.00269 |
| **Clay loam** | 0.22048 | 0.04954 | 0.00027 | 0.16970 | 0.04299 | -0.00003 | 0.11050 | 0.01218 | 0.00226 |
| **Loam** | 0.18896 | 0.04704 | 0.00006 | 0.13353 | 0.03881 | 0.00004 | 0.07247 | 0.00855 | 0.00202 |
| **Loamy sand** | 0.14184 | 0.04258 | -0.00012 | 0.08420 | 0.03136 | 0.00030 | 0.03119 | 0.00459 | 0.00167 |
| **Sand** | 0.08786 | 0.03864 | -0.00008 | 0.04657 | 0.02402 | 0.00073 | 0.01735 | 0.00400 | 0.00130 |
| **Sandy clay** | 0.19501 | 0.04850 | 0.00016 | 0.15614 | 0.04117 | 0.00007 | 0.11436 | 0.01306 | 0.00208 |
| **Sandy clay loam** | 0.16294 | 0.04585 | -0.00003 | 0.12016 | 0.03678 | 0.00017 | 0.07814 | 0.00989 | 0.00182 |
| **Sandy loam** | 0.13762 | 0.04353 | -0.00012 | 0.09180 | 0.03266 | 0.00033 | 0.05067 | 0.00729 | 0.00163 |
| **Silty clay** | 0.29731 | 0.05432 | 0.00081 | 0.24768 | 0.04912 | 0.00000 | 0.18173 | 0.01923 | 0.00276 |
| **Silty clay loam** | 0.26961 | 0.05259 | 0.00063 | 0.21262 | 0.04707 | -0.00003 | 0.13181 | 0.01360 | 0.00265 |
| **Silty loam** | 0.23376 | 0.04991 | 0.00043 | 0.16693 | 0.04326 | 0.00003 | 0.07428 | 0.00745 | 0.00241 |

## Bulk density

For use as a soil parameter in APSIM (as opposed as an input to Equation S1), the effect of SOC on bulk density was based on the mechanistic approach of Adams (1973);

BD = [(SOMf/BD_SOM_) + ((1-SOMf)/BD_Min_)]^-1^, (S3)

where, BD is the bulk density (Mg m^-3^), SOMf is the soil organic matter fraction (g g^-1^), BD_SOM_ is the BD of the soil organic matter (= 0.224), BD_Min_ is the BD of the soil minerals. The value of BD_Min_ is calculated from the initial measured values of BD (BD_t=0_) and SOM (SOMf_t=0_):

BD_Min_ = (1 – SOMf_t=0_)/((1/BD_t=0_) – (SOMf_t=0_/BD_SOM_)). (S4)

## Saturation water content

The water content at saturation (θ_SAT_, m^3^ m^-3^) was calculated from Dalgliesh and Foale (1998),

θ_SAT_= 0.95 (1-BD/2.65), (S5)

where, 2.65 is the assumed particle density of the soil (Mg m^-3^).

## Saturated hydraulic conductivity

The effect of SOC on KSat (mm d^-1^) was based on the approach of Saxton and Rawls (2006):

KSat = α(θ_SAT, t_ - θ_DUL, t_)^(3-1/B)^ , (S6)

B = [ln(ψ_LL_) - ln(ψ_DUL_)]/[ln(θ_DUL, t_) - ln(θ_LL, t_)], (S7)

where, θ_DUL_ is the water content at DUL, θ_LL15_ is the water content at LL15, ψ_DUL_ is the suction at which DUL occurs, ψ_LL_ is the suction at which LL15 occurs and α is an empirical constant. The value of α can be calculated from values of the parameters in Equations S6 and S7 measured at some time (t=0) from,

α = KSat_t=0_ [(θ_SAT, t=0_ - θ_DUL, t=0_)^(3-1/Bt=0)^]^-1^. (S8)

# Estimation of the potential increase of SOC for the Balcarce, New Delhi, Wageningen, and Pendleton sites

To estimate the potential increase of SOC in wheat-based cropping systems for the Balcarce, New Delhi, Wageningen, and Pendleton sites, simulations were undertaken of a management system likely to increase SOC, such as application of manure application or intensification of cropping. This approach follows that used by Luo et al. (2014) who studied potential SOC under grains cropping systems in Australia. The management systems simulated were developed based on published information of the different management practices that may be feasible at each location. Scenarios were simulated for 1000 years and SOC content in the top 0.3 m of soil (three by 0.1 m layers) at the end of the simulation considered to be the maximum achievable increase. Climate data for the 1000 years were based on the available data (e.g. for Pendleton climate data were available from 1930 to 2010), and this data was replicated for the following period of the simulation (up to 1000 years).

In Argentina, manure in combination with 50 percent of conventional rate of inorganic fertiliser was found to be an effective method to increase SOC (Ciapparelli and García, 2014). Thus for the Balcarce site, the wheat cropping system previously simulated (Asseng et al., 2013) was modified to include an application of 16 Mg ha^-1^ of manure with a C:N ratio of 20 applied annually 10 days prior to sowing. Manure was 90 percent incorporated to a depth of 0.15 m with a tillage operation. Nitrogen fertiliser application rate was reduced from the original rate of 120 kg N ha^-1^ to 60 kg N ha^-1^.

In India, a long-term experiment in New Delhi found the biannual application of manure to a maize, wheat, cowpea biannual rotation agro-ecosystem increased SOC (Kanchikerimath and Singh, 2001; Rudrappa et al., 2006). For the New Delhi simulation, the wheat-based biannual cropping system previously simulated (Asseng et al., 2013) was intensified to an annual cropping system with maize and cowpea crops added to the rotation and stubble retention to maximise SOC sequestration (pers. comm. Balwinder Singh, 2015). Manure with a C:N ratio was applied annually at a rate of 15 Mg ha^-1^. The manure C:N ratio was based on the average C:N ratio for Indian manure (Dalal et al., 2003). Manure was 90 percent incorporated to a depth of 0.15 m with a tillage operation. A maize cultivar (Ekka_Kaveri_2012) already parameterised and included in the APSIM-Maize module was selected as appropriate for the region (pers. comm. Don Gaydon 2015), with 140 kg N ha^-1^ of N fertiliser applied annually. The cowpea cultivar Banjo was selected as appropriate for the region (pers. comm. Don Gaydon 2015). For the wheat crop, the irrigation schedule for the original simulations was retained (total of 385 mm applied). For the maize and cowpea crops, 75 mm of water was applied at sowing as per the amounts applied in the experiment described by (Kanchikerimath and Singh, 2001; Rudrappa et al., 2006).

In the Netherlands, applying manure is an effective method to increase SOC. However, legislation restricts the application of livestock manure to an agricultural agro-ecosystem to an amount that corresponds to 170 kg N ha^-1^ yr^-1^ (European Commission, 2010). This amount of nitrogen is equivalent to the addition of 8.5 Mg ha^‑1^ of manure with a C:N ratio of 20. This amount and quality of manure was added annually four days prior to sowing to the wheat cropping system previously simulated (Asseng et al., 2013). Manure was 90 percent incorporated to a depth of 0.15 m with a tillage operation. In addition, 71 kg N ha^‑1^ of fertiliser was added annually.

In the low precipitation dryland cereal cropping regions of the United States of America, crops are often grown biannually. However, although more risky, annual cropping can have positive environmental benefits (Schillinger et al., 2008). For the Pendleton site, cropping intensification with stubble retention was utilised to increase SOC. An annual, rather than a biannual wheat crop was simulated. Tillage operations were decreased from four operations to one, to reflect the reduced time in which weeds need to be controlled in the annual cropping system. In addition, 90 kg N ha^‑1^ of fertiliser was added annually.

# Model testing for the Pendleton site

## Experiment site

The Pendleton Residue Management experiment site is located near Pendleton, Oregon in the United States of America. Nine treatments, with two replicates per treatment, were applied to a winter wheat / fallow rotation from 1931 (Table S6). A medium-tall soft white variety of wheat was grown from 1931 to 1966 and a semi-dwarf varieties from 1967-1986. Further information about the site is provided by Rasmussen et al. (1998a, b).

**Table S6.** Details of treatments at the Pendleton site.

|  | **Straw management** | | | **Organic residue addition** | | | **Inorganic N fertiliser (kg ha^-1^)** | | |
| --- | --- | --- | --- | --- | --- | --- | --- | --- | --- |
| **Treatment code** | 1931-1966 | 1967-1978 | 1979-1986 | 1931-1966 | 1967-1978 | 1979-1986 | 1931-1966 | 1967-1978 | 1979-1986 |
| **fB-N0** | Fall Burn | Fall Burn | Fall Burn | 0 | 0 | 0 | 0 | 0 | 0 |
| **sB-N0** | Spring Burn | Spring Burn | Spring Burn | 0 | 0 | 0 | 0 | 0 | 0 |
| **nB-N0** | No Burn | No Burn | No Burn | 0 | 0 | 0 | 0 | 0 | 0 |
| **sB-N45** | Fall Disc | No Burn | Spring Burn | 0 | 0 | 0 | 0 | 45 | 45 |
| **sB-N90** | Spring Disc | No Burn | Spring Burn | 0 | 0 | 0 | 0 | 90 | 90 |
| **nB-N45** | Fall Disc | No Burn | No Burn | 0 | 0 | 0 | 34 | 45 | 45 |
| **nB-N90** | Spring Disc | No Burn | No Burn | 0 | 0 | 0 | 34 | 90 | 90 |
| **nB-PV** | No Burn | No Burn | No Burn | Pea Vines = 2.24 wet t/ha | | | 0 | 0 | 0 |
| **nB-MN** | No Burn | No Burn | No Burn | Manure = 22.4 wet t/ha | | | 0 | 0 | 0 |

## Model parameterisation

APSIM (v.7.7) soil N, soil carbon, soil water, surface organic matter, and crop modules were parameterised using measurements (Rasmussen et al., 1998a, b) or default values (Table S7). Initial water was set at 100 % of the soil profile. Initial root weight was 1109 kg ha^-1^ with a C:N ratio of 57. The soil C:N ratio was 13. Initial surface residue was 1000 kg ha^-1^ of wheat stubble with a C:N ratio of 80. Data from 1931-1986 was used for this analysis, with the simulation commencing in 1930.

**Table S7.** Soil and water values used to parameterise APSIM to the Pendleton site.

| **Soil depth (m)** | **LL15**  **(mm mm^-1^)^a^** | **DUL**  **(mm mm^-1^)** | **Saturati-on (mm mm^-1^)** | **Bulk density (Mg m^-3^)** | **Saturated hydraulic conductivity (mm**  **day^-1^)** | **Wheat KL (/day)** | **Wheat XF**  **(0-1)** | **SWC**  **-ON**  **(0-1)** | **Organic carbon (Total %)** | **FBiom (0-1)** | **Finert (0-1)** | **Initial nitrate (kg ha^-1^)** | **Initial ammonium (kg ha^-1^)** |
| --- | --- | --- | --- | --- | --- | --- | --- | --- | --- | --- | --- | --- | --- |
| **0.0-0.15** | 0.110 | 0.320 | 0.520 | 1.240 | 562.000 | 0.06 | 1.0 | 0.500 | 1.420 | 0.040 | 0.350 | 10.200 | 0.000 |
| **0.15-0.3** | 0.110 | 0.320 | 0.520 | 1.250 | 562.000 | 0.06 | 1.0 | 0.500 | 1.200 | 0.030 | 0.500 | 19.900 | 0.000 |
| **0.3-0.6** | 0.110 | 0.310 | 0.510 | 1.260 | 557.000 | 0.05 | 1.0 | 0.500 | 0.950 | 0.020 | 0.650 | 19.000 | 0.000 |
| **0.6-0.9** | 0.100 | 0.310 | 0.500 | 1.290 | 959.000 | 0.04 | 1.0 | 0.500 | 0.500 | 0.020 | 0.750 | 15.500 | 0.000 |
| **0.9-1.2** | 0.100 | 0.300 | 0.490 | 1.300 | 1063.000 | 0.04 | 1.0 | 0.500 | 0.225 | 0.015 | 0.850 | 21.100 | 0.000 |
| **1.2-1.5** | 0.100 | 0.300 | 0.490 | 1.300 | 1063.000 | 0.02 | 0.0 | 0.500 | 0.150 | 0.010 | 0.990 | 17.700 | 0.000 |
| **1.5-1.8** | 0.100 | 0.300 | 0.490 | 1.300 | 1063.000 | 0.01 | 0.0 | 0.500 | 0.150 | 0.010 | 0.990 | 17.700 | 0.000 |

^a^ Crop lower limit was set to soil LL15.

Climate data primarily came from an on-site meteorological station. When these data were not available, data were taken from nearby meteorological stations including the Pendleton Bridge Experimental station, Pendleton Downton, Pendleton Regional Airport and Pilot Rock. Any missing data in these records were in-filled with long-term daily averages.

Simulated winter wheat was planted on 15 October and harvested the following July, after which there was a fallow of approximately 13.5 months. Simulated tillage operations reflected general tillage operations at the site (Table S8).

**Table S8.** Tillage operations for the Pendleton site.

| **Date** | **Tillage operation** | **Fraction of reside incorporated (0-1)** | **Tillage depth (mm)** |
| --- | --- | --- | --- |
| **16 May** | Moldboard plough | 0.95 | 200 |
| **16 June** | RodWeed row plant | 0.2 | 60 |
| **16 July** | RodWeed row plant | 0.2 | 60 |
| **16 August** | RodWeed row plant | 0.2 | 60 |

Two wheat varieties (Pendleton_tall and Pendleton_dwarf) were simulated using default varieties with modifications to reflect on-site measurements (Table S9).

**Table S9.** Variables modified from default wheat variety values to reflect the varieties grown at the Pendleton site.

| **Variable** | **Pendleton_tall**  **1931 - 1966** | **Pendlton_dwarf 1967 - 1986** |
| --- | --- | --- |
| **tt_start_grain_fill** | 602 | 602 |
| **tt_end_grain_fill** | 38 | 38 |
| **startgf_to_mat** | 640 | 640 |
| **vern_sens** | 5 | 5 |
| **photop_sens** | 5 | 5 |
| **grains_per_gram_stem** | 20 | 27 |
| **potential_grain_filling_rate** | 0.0028 | 0.028 |
| **potential_grain_growth_rate** | 0.001 | 0.001 |
| **max_grain_size** | 0.045 | 0.045 |

## Results

Simulated average grain yields were closer to the measured yields for the semi-dwarf variety (r^2^ = 0.90; Figure S3) than for the medium tall variety (r^2^ = 0.69). Simulated average grain yields tended to be under predicted for the semi-dwarf variety and over predicted for the medium tall variety.

Simulated average straw yields were closer to the measured straw yields for the medium tall variety (r^2^ = 0.82; Figure S4) than for the semi-dwarf variety (r^2^ = 0.56). Simulated average straw yields were similar to the grain yield in that they tended to be under predicted for the semi-dwarf variety and over predicted for the medium tall variety.

Average N uptake tended to be over predicted for both the medium tall and the semi-dwarf varieties (Figure S5).

Soil organic carbon was well simulated over time in the 0-0.3 m soil layer. However, it tended to be over predicted in the 0.3-0.6 m soil layer towards the end of the simulation period (Figure S6).

Simulated total soil N was well simulated over time in the 0-0.3 m soil layer, however, it was consistently under predicted in the 0.3-0.6 m soil layer (Figure S7).

**
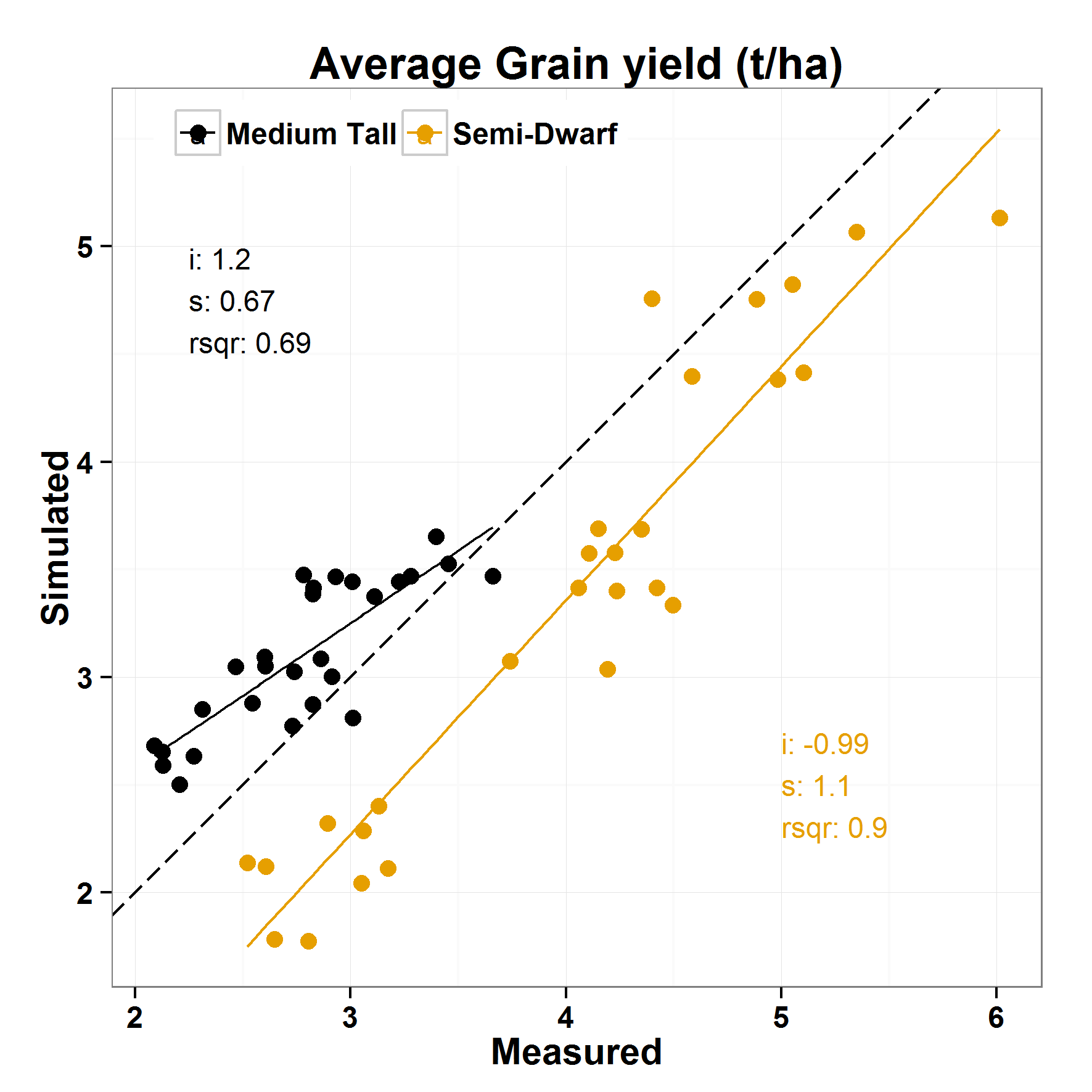
**

**Figure S3.** Measured and simulated average grain yield for the medium tall and semi-dwarf varieties for the Pendleton site. Average grain yield represents the mean grain yield for each treatment for a 10 year time period.

**
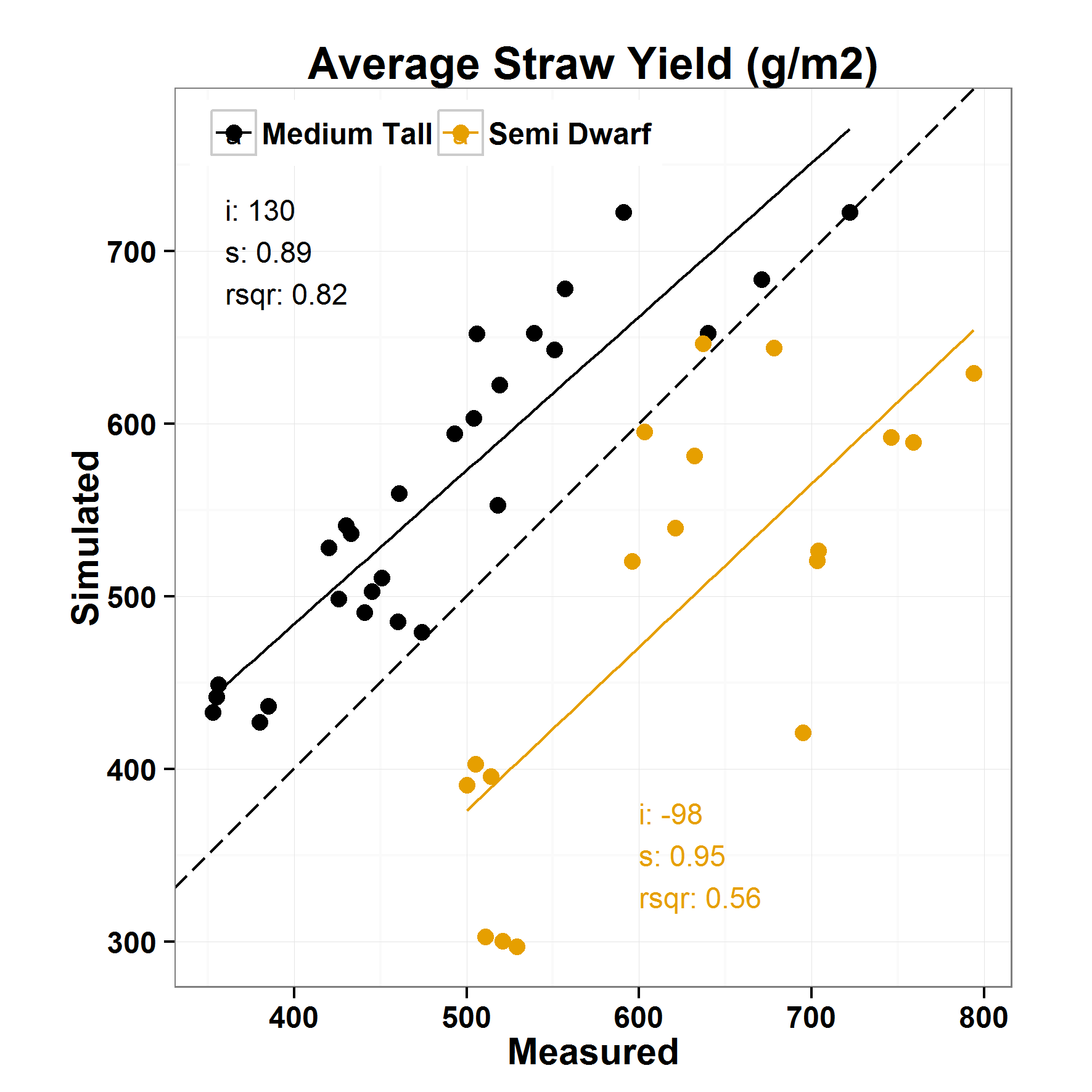
**

**Figure S4.** Measured and simulated average straw yield for the medium tall and semi-dwarf varieties for the Pendleton site. Average straw yield represents the mean straw yield for each treatment for a 10 year time period.

**
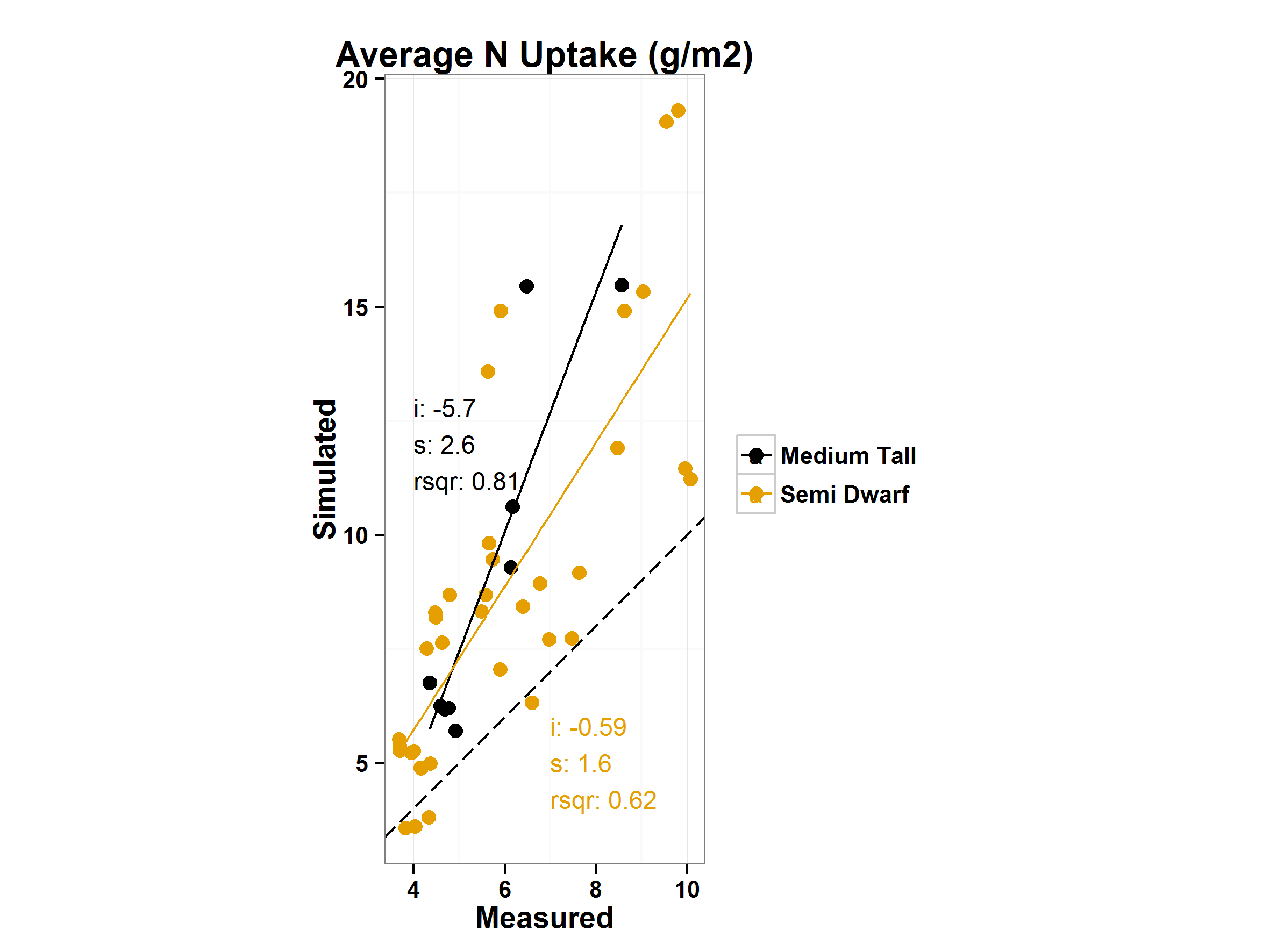
**

**Figure S5.** Measured and simulated average N uptake for the medium tall and semi-dwarf varieties for the Pendleton site. Average N uptake represents the mean N uptake for each treatment for a 10 year time period.

**
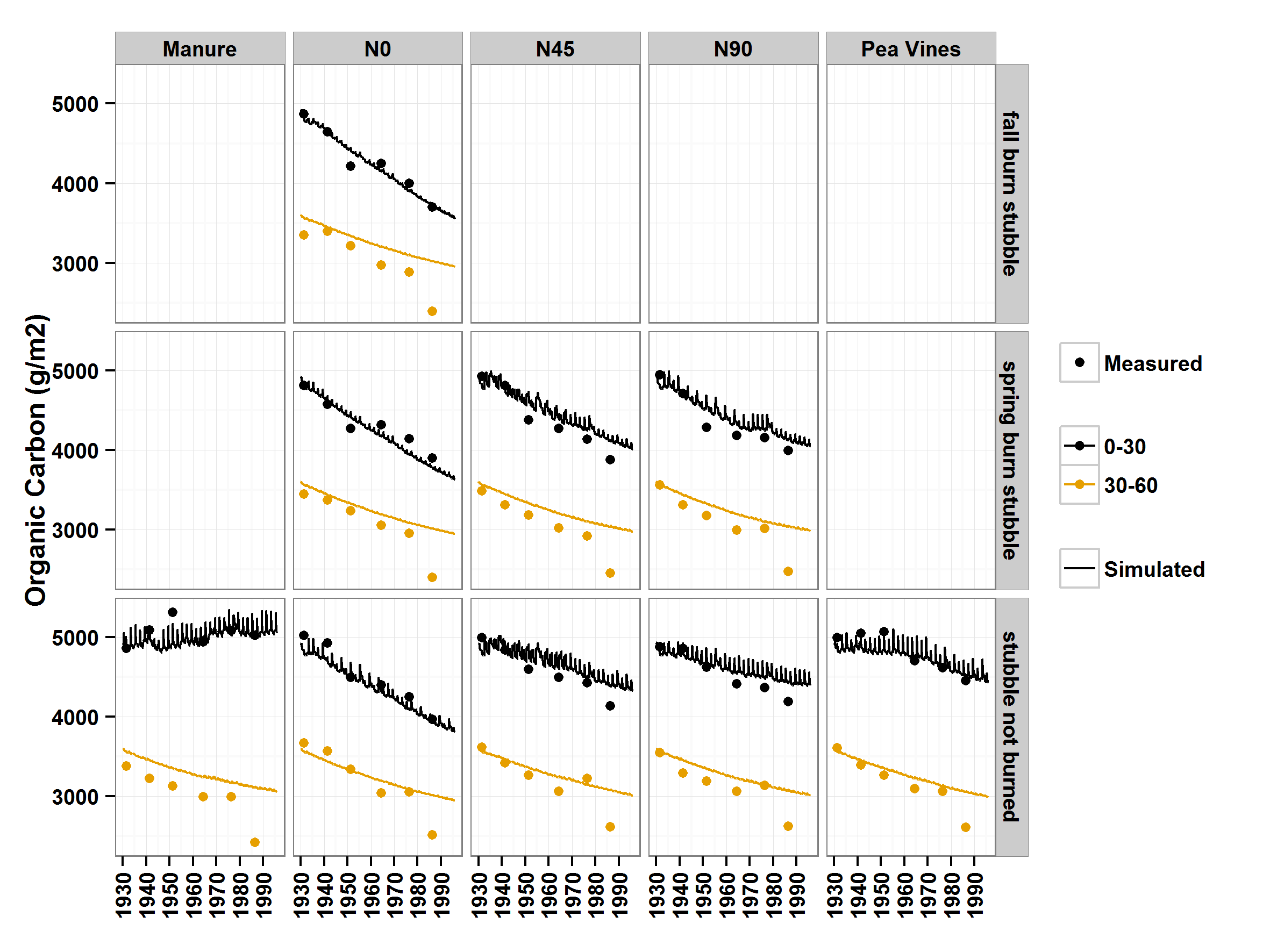
**

**Figure S6.** Measured (points) and simulated (line) organic carbon for 0-0.3 m (black) and 0.3-0.6 m (yellow) soil layers for the Pendleton site.

**
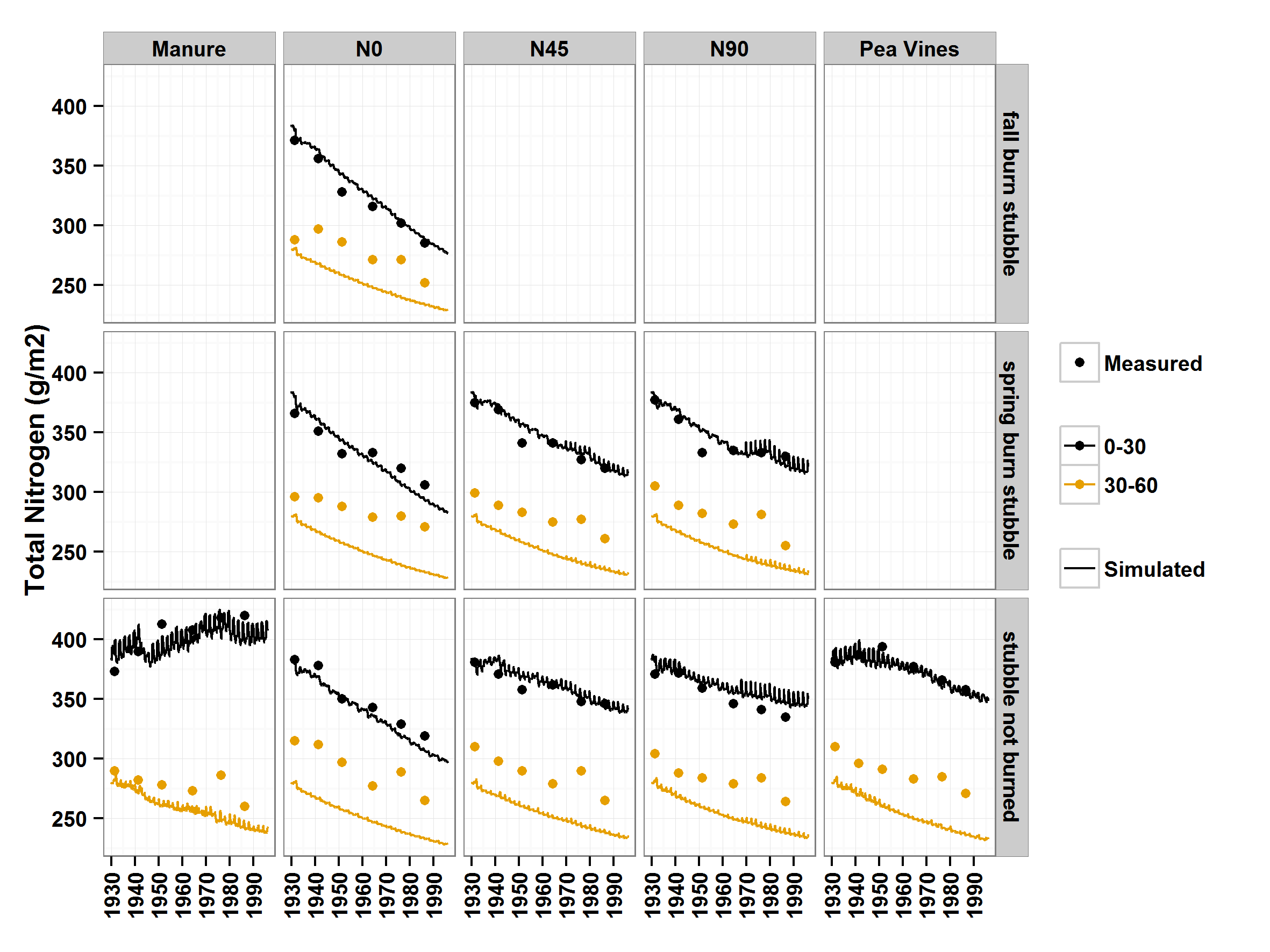
**

**Figure S7.** Measured (points) and simulated (line) total N for 0-0.3 m (black) and 0.3-0.6 m (yellow) soil layers for the Pendleton site.

# References

Adams, W. A. (1973). The effect of organic matter on the bulk and true densities of some uncultivated podzolic soils. *J. Soil Sci.* 24, 10–17. doi:10.1111/j.1365-2389.1973.tb00737.x.

Alexander, E. B. (1980). Bulk densities of California soils in relation to other soil properties. *Soil Sci. Soc. Am. J.* 44, 689–692.

Asseng, S., Ewert, F., Martre, P., Rotter, R. P., Lobell, D. B., Cammarano, D., et al. (2013). Uncertainty in simulating wheat yields under climate change. *Nat. Clim. Chang.* 3, 627–632. doi:10.1038/ncliamte1916.

Bernoux, M., Cerri, C., Arrouays, D., Jolivet, C., and Volkoff, B. (1998). Bulk Densities of Brazilian Amazon Soils Related to Other Soil Properties. *Soil Sci. Soc. Am. J.* 62, 743. doi:10.2136/sssaj1998.03615995006200030029x.

Ciapparelli, I. C., and García, A. R. (2014). Use of manure to wheat production in an Argentinean Hapludoll soil. *J. Pollut. Eff. Control* 3, 1–4. doi:10.4172/2375-4397.1000131.

Cichota, R., Vogeler, I., Snow, V. O., and Webb, T. H. (2013). Ensemble pedotransfer functions to derive hydraulic properties for New Zealand soils. *Soil Res.* 51, 94-111. doi:10.1071/SR12338.

Dalal, R. C., Acharya, C. L., Reddy, K. S. Redding, M., Sharma, A. K., Ritchie, J. et al. (2003). Survey of potential manure for meeting crop nutrient needs with integrated nutrient management in Madhya Pradesh, India. ICAR-ACIAR project final report.

Dalgliesh, N., and Foale, M. (1998). Soil matters: Monitoring soil water and nutrients in dryland farming. Toowoomba: CSIRO.

European Commission (2010). The EU Nitrates Directive. Available online at: http://ec.europa.eu/environment/pubs/pdf/factsheets/nitrates.pdf.

Gupta, S. C., and Larson, W. E. (1979). Estimating soil water retention characteristics from particle size distribution, organic matter percent, and bulk density. *Water Resour. Res.* 15, 1633–1635. doi:10.1029/WR015i006p01633.

International Society of Soil Science (1929). Minutes of the first commission meetings, International Congress of Soil Science. *Transactions of the First Commission of the International Society of Soil Science*. 4, 215-220.

Kanchikerimath, M., and Singh, D. (2001). Soil organic matter and biological properties after 26 years of maize – wheat – cowpea cropping as affected by manure and fertilization in a Cambisol in semiarid region of India. *Agric. Ecosyst. Environ.* 86, 155–162. doi:10.1016/S0167-8809(00)00280-2.

Krull, E. S., Skjemstad, J. O., and Baldock, J. A. (2004). Functions of soil organic matter and the effect on soil properties. Glen Osmond.

Luo, Z., Wang, E., Baldock, J., and Xing, H. (2014). Potential soil organic carbon stock and its uncertainty under various cropping systems in Australian cropland. *Soil Res.* 52, 463–475. doi:10.1071/SR13294.

Manrique, L. A., and Jones, C. A. (1991). Bulk density of soils in relation to soil physical and chemical properties. *Soil Sci. Soc. Am. J.* 55, 476-481. doi:10.2136/sssaj1991.03615995005500020030x.

Minasny, B., and Hartemink, A. E. (2011). Predicting soil properties in the tropics. *Earth-Science Rev.* 106, 52–62. doi:10.1016/j.earscirev.2011.01.005.

Minasny, B., and McBratney, A. B. (2001). The Australian soil texture boomerang: A comparison of the Australian and USDA/FAO soil particle-size classification systems. *Aust. J. Soil Res.* 39, 1443–1451. doi:10.1071/SR00065.

Murphy, B. W. (2015). Impact of soil organic matter on soil properties — a review with emphasis on Australian soils. *Soil Res.* 53, 605–635. doi:10.1071/SR14246.

Nelson, D. W., and Sommers, L. E. (1982). “Total carbon, organic carbon and organic matter,” in *Methods of Soil Analysis: Part 2 Chemical and Microbiological Properties*, eds. A. L. Page, R. H. Miller, and D. R. Keeny (Madison: Soil Science Society of America), 539-579.

Northcote, K. H. (1971). *A factual key for the recognition of Australian soils*. 3rd edn., Glenside: Rellim Technical Publications.

Pidgeon, J. D. (1972). The measurement and prediction of available water capacity of ferrallitic soils in Uganda. *J. Soil Sci.* 23, 432–441. doi:10.1111/j.1365-2389.1972.tb01674.x.

Probert, M. E., Dimes, J. P., Keating, B. A., Dalal, R. C., and Strong, W. M. (1998). APSIM’s water and nitrogen modules and simulation of the dynamics of water and nitrogen in fallow systems. *Agric. Syst.* 56, 1–28. doi:10.1016/S0308-521X(97)00028-0.

Rasmussen, P. E., Albrecht, S. L., and Smiley, R. W. (1998a). Soil C and N changes under tillage and cropping systems in semi-arid Pacific Northwest agriculture. *Soil Tillage Res.* 47, 197–205. doi:10.1016/S0167-1987(98)00106-8.

Rasmussen, P. E., Goulding, K. W. T., Brown, J. R., Grace, P. R., Janzen, H., and Korschens, M. (1998b). Long-term agroecosystem experiments: Assessing agricultural sustainability and global change. *Science.* 282, 893–896. doi:10.1126/science.282.5390.893.

Rawls, W. J., Ahuja, L. R., and Brakensiek, D. L. (1992). “Estimating soil hydraulic properties from soils data,” in *Indirect methods for estimating the hydraulic properties of unsaturated soils,* eds. M. Th. van Genuchten, and F. J. Leij, Proceedings of the international workshop on indirect methods for estimating the hydraulic properties of unsaturated soils (Riverside: California), 329-341.

Rudrappa, L., Purakayastha, T. J., Singh, D., and Bhadraray, S. (2006). Long-term manuring and fertilization effects on soil organic carbon pools in a Typic Haplustept of semi-arid sub-tropical India. *Soil Tillage Res.* 88, 180–192. doi:10.1016/j.still.2005.05.008.

Saxton, K., and Rawls, W. (2006). Soil water characteristic estimates by texture and organic matter for hydrologic solutions. *Soil Sci. Soc. Am. J.* 70, 1569–1578. doi:10.2136/sssaj2005.0117.

Schillinger, W. F., Schofstoll, S. E., and Alldredge, J. R. (2008). Available water and wheat grain yield relations in a Mediterranean climate. *F. Crop. Res.* 109, 45–49. doi:10.1016/j.fcr.2008.06.008.

Spouncer, L. R., Skjemstad, J. O., and Merry, R. H. (2000). Soil carbon information for major soils in IBRA regions.

Tomasella, J., and Hodnett, M. G. (1998). Estimating soil water retention characteristics from limited data in Brazilian Amazonia. *Soil Sci.* 163, 190-202. doi: 10.1097/00010694-199803000-00003.

Tomasella, J., Hodnett, M. G., and Rossato, L. (2000). Pedotransfer functions for the estimation of soil water retention in Brazilian soils. *Soil Sci. Soc. Am. J.* 64, 327–338. doi:10.2136/sssaj2000.641327x.

Tranter, G., Minasny, B., Mcbratney, A. B., Murphy, B., Mckenzie, N. J., Grundy, M., et al. (2007). Building and testing conceptual and empirical models for predicting soil bulk density. *Soil Use Manag.* 23, 437–443. doi:10.1111/j.1475-2743.2007.00092.x.

Vereecken, H., Maes, J., Feyen, J., and Darius, P. (1989). Estimating the soil moisture retention characteristic from texture, bulk density, and carbon content. *Soil Sci.* 148, 389–403. doi: 0038-075X/89/1486-0389.

Weynants, M., Vereecken, H., and Javaux, M. (2009). Revisiting Vereecken Pedotransfer Functions: Introducing a Closed-Form Hydraulic Model. *Vadose Zo. J.* 8, 86-95. doi:10.2136/vzj2008.0062.

Williams, J., Ross, P., and Bristow, K. (1992). “Prediction of the Campbell water retention function from texture, structure and organic matter,” in *Indirect methods for estimating the hydraulic properties of unsaturated soils,* eds. M. Th. van Genuchten, and F. J. Leij, Proceedings of the international workshop on indirect methods for estimating the hydraulic properties of unsaturated soils (Riverside: California), 427-441.

Wosten, J. H. M., Lilly, A., Nemes, A., and Le Bas, C. (1999). Development and use of a database of hydraulic properties of European soils. *Geoderma* 90, 169–185. doi:10.1016/S0016-7061(98)00132-3.

Wösten, J. H. M., Pachepsky, Y. A., and Rawls, W. J. (2001). Pedotransfer functions: bridging the gap between available basic soil data and missing soil hydraulic characteristics. *J. Hydrol.* 251, 123–150. doi:10.1016/S0022-1694(01)00464-4.
